# Accommodating detection limits of multiple exposures in environmental mixture analyses: an overview of statistical approaches

**DOI:** 10.1186/s12940-024-01088-w

**Published:** 2024-05-16

**Authors:** Myeonggyun Lee, Abhisek Saha, Rajeshwari Sundaram, Paul S. Albert, Shanshan Zhao

**Affiliations:** 1 Biostatistics and Computational Biology Branch, National Institute of Environmental Health Sciences, National Institutes of Health, Durham, NC USA; 2 Biostatistics and Bioinformatics Branch, Division of Population Health Research, Eunice Kennedy Shriver National Institute of Child Health and Human Development, National Institutes of Health, Bethesda, MD USA; 3 Biostatistics Branch, Division Cancer Epidemiology and GeneticsBiostatistics Branch, Division of Cancer Epidemiology and Genetics, National Cancer Institute, National Institutes of Health, Bethesda, MD USA

**Keywords:** Left-censored exposures, Detection limits, Environmental mixtures, Imputation

## Abstract

**Background:**

Identifying the impact of environmental mixtures on human health is an important topic. However, such studies face challenges when exposure measurements lie below limit of detection (LOD). While various approaches for accommodating a single exposure subject to LOD have been used, their impact on mixture analysis has not been thoroughly investigated. Our study aims to understand the impact of five popular LOD accommodation approaches on mixture analysis results with multiple exposures subject to LOD, including omitting subjects with any exposures below LOD (complete case analysis); single imputations by LOD/$\sqrt{2}$, and by estimates from a censored accelerated failure time (AFT) model; and multiple imputation (MI) with or without truncation based on LOD.

**Methods:**

In extensive simulation studies with high-dimensional and highly correlated exposures and a continuous health outcome, we examined the performance of each LOD approach on three mixture analysis methods: elastic net regression, weighted quantile sum regression (WQS) and Bayesian kernel machine regression (BKMR). We further analyzed data from the National Health and Nutrition Examination Survey (NHANES) on how persistent organic pollutants (POPs) influenced leukocyte telomere length (LTL).

**Results:**

Complete case analysis was inefficient and could result in severe bias for some mixture methods. Imputation by LOD/$\sqrt{2}$ showed unstable performance across mixture methods. Conventional MI was associated with consistent mild biases, which can be reduced by using a truncated distribution for imputation. Estimating censored values by AFT models had a minimal impact on the results. In the NHANES analysis, imputation by LOD/$\sqrt{2}$, truncated MI and censored AFT models performed similarly, with a positive overall effect of POPs on LTL while PCB126, PCB169 and furan 2,3,4,7,8-pncdf being the most important exposures.

**Conclusions:**

Our study favored using truncated MI and censored AFT models to accommodate values below LOD for the stability of downstream mixture analysis.

**Supplementary Information:**

The online version contains supplementary material available at 10.1186/s12940-024-01088-w.

## Background

Environmental exposures to chemical, biological, or physical substances found in air, water, food, or soil are common during the human life course [1–3]. These high-dimensional and highly correlated exposures can act synergistically or antagonistically on human health [4, 5]. Studying individual exposures only addresses their marginal effects, without accounting for others, which can result in misleading conclusions about effects of the whole mixture [6, 7].

Several popular modeling approaches exist to analyze complex environmental mixtures, including but not limited to regularized regressions, weighted quantile sum regression (WQS) [8, 9], and Bayesian kernel machine regression (BKMR) [10, 11]. Briefly, regularized regressions such as elastic net regression [12] and lasso (least absolute shrinkage and selection operator) [13] can be used to identify the relative importance of driver(s) in the mixture through variable selection [14–16]. WQS derives a one-dimensional weighted sum score of the exposures with a linear relationship to a continuous health outcome under the assumption that all exposure effects are in the same direction. WQS has been generalized to several types of outcomes [17] and is widely used in practice [16, 18–20]. BKMR is a Bayesian nonparametric method to handle complex nonlinear relationships between exposure mixtures and continuous, binary, and time-to-event outcomes [10, 21]. It has been widely used in mixtures studies, due to its flexibility and abundant visualization tools [16, 22]. Details of these methods are illustrated in Appendix A.

Environmental health studies often face challenges of exposure values below limit of detection (LOD) (i.e., left-censored). All the above-mentioned mixture methods assume accurate measurements of exposures, thus some procedure for accommodating LOD is needed before applying these mixture methods. For example, with data from the National Health and Nutrition Examination Survey (NHANES) 2001–2002 cycle, Gibson et al. [23] investigated the relationship between persistent organic pollutants (POPs) and leukocyte telomere length (LTL), a biomarker associated with chronic diseases [24–27] and dioxin-associated cancers [28–31]. Among the 34 POPs with 1.4% to 99.9% of values below LOD [32], Gibson et al. [23] restricted their analysis to the 18 POPs with less than 40% of values below LOD and imputed all values below LOD by LOD/$\sqrt{2}$. However, it is unclear how this imputation influenced the analysis results.

Recovering the true effects of environmental mixtures, where multiple exposures are subject to different proportions of values below LOD, is thus an important problem to address. Several approaches for accommodating values below LOD for a single exposure have been used in practice, including complete case analysis by omitting subjects with any measured values below LOD, single imputation by LOD/$\sqrt{2}$, and multiple imputation. Ortega-Villa et al. [33] empirically compared these approaches in an environmental study setting with a binary outcome and a single exposure. However, the impact of these LOD approaches on downstream mixture analysis results has not been thoroughly investigated in settings where multiple exposures within the high-dimensional and highly correlated exposure mixtures are subject to LOD.

In this manuscript, we aim to understand the impact of five popular approaches for accommodating LOD: complete case analysis; single imputation of values below LOD by LOD/$\sqrt{2}$ and by estimates from censored accelerated failure time (AFT) models; and multiple imputation (MI) with and without LOD-based truncation. We conducted extensive simulation studies to examine their influences on three popular mixture methods, including elastic net regression, WQS and BKMR, after applying the above-mentioned LOD approaches. We also re-analyzed the 2001–2002 NHANES dataset as described in Gibson et al. [23], to illustrate how different ways of handling LOD can impact the identification of associations between the POPs and LTL. Through these simulated and real data examples, we would like to draw readers’ attention to carefully choose LOD accommodation approaches for mixture analysis, rather than recommending one approach as the gold standard.

## Methods

### LOD accommodation approaches

Here we give a brief review of the five LOD accommodation approaches. Complete case analysis only includes subjects whose exposure values are all above LOD. In theory, this approach provides unbiased results for linear regressions when the missingness only depends on the exposures [34, 35]. However, its performance may be unstable in practice with reduced sample sizes [36, 37]. An alternative approach is to replace values below LOD with a pre-specified constant value such as LOD, LOD/2, or LOD/$\sqrt{2}$ based on the observed exposure distribution [37–39]. In this study we chose LOD/$\sqrt{2}$, which is widely used for log-normally distributed (or right skewed) chemical exposures. This approach is popular due to its simplicity, but results may be biased when the distribution of values below LOD is not centered on the substitution value [37, 38, 40].

Chen et al. [41] recently proposed a new approach using multivariate accelerated failure time (AFT) regressions to model multiple left-censored chemicals through baseline covariates, which is a flexible approach specialized to handle censored outcomes with mild assumptions about the joint distribution. Note that this is an extension of the approach proposed in Kong and Nan [42] from a single exposure subject to LOD to multiple exposures. Due to simultaneously fitting multiple AFT models, it allows one to specifically account for the correlations between chemicals through shared baseline covariates and correlation between error terms. The originally proposed approach in Chen et al. [41] allows one to simultanueous model the exposures and health outcomes with efficiency gain. However, due to practical considerations, we only adopted the first part of this approach with the multivariate AFT model to conduct a single imputation for simplicity. The details of this approach are described in Appendix B.

Lastely, instead of single imputation approaches described above, multiple imputation (MI) also has been widely used by treating values below LOD as missing, and then imputing with models such as Bayesian linear regression or linear regression with bootstrap samples [35, 43]. MI generates multiple datasets (e.g., 5 or 10) for downstream analysis and combine analysis results using the Rubin’s rule [44]. In this study we chose to use the bootstrap linear regression implemented in the R ‘mice’ package [45] due to its superior performance in the settings we investigated. However, conventional MI does not guarantee that imputed values are below LOD. Thus, we improved it by truncating the estimated normal distribution at LOD to ensure all imputed values are in the correct range, and named this approach as truncated MI. The details of conventional and truncated MI are described in Appendix C.

### Simulation settings

We conducted extensive simulations to empirically evaluate the impact of LOD accommodation approaches on three popular downstream mixture analysis methods, including elastic net regression, WQS and BKMR, under various settings. First, covariates $X={\left(1, {X}_{1},{X}_{2}\right)}^{T}$ were independently generated from ${X}_{1} \sim Bern\left(p=0.5\right)$ and ${X}_{2} \sim N\left(1, 1\right)$. Given that environmental exposures are commonly highly correlated, right-skewed and associated through covariates, a mixture of $p=10$ exposures $Z={\left({Z}_{1},\dots , {Z}_{10}\right)}^{T}$ was generated from a multivariate linear regression model with covariates $X$ and log link, that is, ${Z}_{log}={\text{log}}\left(Z\right)={\eta }^{T}X+\xi$, with $$\eta =\left[{\eta }_{1},\dots , {\eta }_{10}\right]=\left[\begin{array}{ccc}0.20& 0.35& \begin{array}{ccc}0.30& 0.25& \begin{array}{ccc}0.35& 0.25& \begin{array}{ccc}0.25& 0.40& \begin{array}{cc}0.25& 0.30\end{array}\end{array}\end{array}\end{array}\\ 0.50& 0.50& \begin{array}{ccc}0.25& 0.05& \begin{array}{ccc}0.03& 0.10& \begin{array}{ccc}0.25& 0.25& \begin{array}{cc}0.50& 0.25\end{array}\end{array}\end{array}\end{array}\\ 0.05& 0.02& \begin{array}{ccc}0.00& 0.50& \begin{array}{ccc}0.25& 0.25& \begin{array}{ccc}0.25& 0.50& \begin{array}{cc}0.25& 0.25\end{array}\end{array}\end{array}\end{array}\end{array}\right],$$ and $\xi \sim MVN\left(0,\Sigma \right)$ with $$\Sigma ={\upsigma }^{2}\left(\begin{array}{ccc}{R}_{1}& 0& 0\\ 0& {R}_{2}& 0\\ 0& 0& {R}_{3}\end{array}\right)$$, where ${R}_{1}$ and ${R}_{2}$ are 3 × 3 correlation matrices with all off-diagonal entries as 0.25 and 0.75, respectively, and ${R}_{3}$ is a 4 × 4 correlation matrix with all off-diagonal entries as 0.5. Through this formulation, we imposed correlations between exposures through two sources: shared covariate effects $X$, where the correlations are governed by $\eta$, and correlation between error terms through off-diagonal entries in $\Sigma$. By the group structure in $\Sigma$ (i.e., $\left\{{Z}_{1},{Z}_{2}, {Z}_{3}\right\}$ for group 1, $\left\{{Z}_{4},{Z}_{5}, {Z}_{6}\right\}$ for group 2, and $\left\{{Z}_{7},{Z}_{8}, {Z}_{9}, {Z}_{10}\right\}$ for group 3), we allowed a higher within-group correlation than between-group correlations. We varied $\sigma =1/2$ and $1/8$ for moderate and high correlations within the groups, respectively (see Figure S1 for Spearman correlation coefficients between simulated variables). Because $Z$ were right-skewed, we generated outcome $Y$ under a linear regression with the log-transformed ${Z}_{log}$, as in many environmental health studies, that is, $Y={\beta }^{T}{Z}_{log}+{\alpha }^{T}X+\epsilon ,$ where $\epsilon \sim N\left(0, 2\right)$. With a sample size of 500, we fixed $\alpha =\left(1, 1, 1\right)$, and varied $\beta$ and percent of value below LOD in various scenarios as follows.

- *Scenario 1.* We set $\beta ={\left(1.0, 0.8, 0.0, 0.6, 0.4, 0.0, 0.2, 0.1, 0.0, 0.0\right)}^{T}$ to reflect the relative importance of these exposures, and assumed that ${Z}_{2},{Z}_{3},{Z}_{5},{Z}_{7}$, and ${Z}_{9}$ have approximately 30% of values below LOD.
- *Scenario 2.* ${Z}_{2}$ is assumed to have approximately 70% of values below LOD, while all the other settings are the same as in Scenario 1. In this scenario, we handled ${Z}_{2}$ in two ways that are widely used in practice: (i) ${Z}_{2}$ was completely excluded from the analysis (Scenario 2A), and (ii) an indicator variable of whether ${Z}_{2}$ is above the LOD was used (Scenario 2B), while the other exposures subject to LOD were handled with the above-mentioned approaches. This scenario allows us to understand how to handle an exposure with a high percent of values below LOD.
- *Scenario 3*. We generated all the exposures as in Scenario 1, but we re-generated a new ${Z}_{2}$ from $Unif\left(0, LOD\right)$ if the original ${Z}_{2}$ was below LOD. This essentially resulted in the marginal distribution of ${Z}_{2}$ being a mixture distribution of uniform below LOD and normal above LOD, and the new ${Z}_{2}$ was used to simulate the outcome $Y$. In this scenario, we aim to investigate whether the LOD accommodation approaches hold when the distributions of exposures are different below and above LOD. In this example, we arbitarily assumed that the change point of distribution was exactly at LOD as a case study. In practice we may not know the change point unless there are external information. All the other settings are the same as in Scenario 1.
- *Scenario 4.* We assumed a null effect (i.e., ${\beta }_{2}=0$) of ${Z}_{2}$ for values below LOD and ${\beta }_{2}=0.8$ for values above LOD. The other settings are the same as those in Scenario 1. This allows us to investigate whether the LOD accommodation approaches hold when the relationships between exposures and outcome are different below and above the LOD. Again, as a case study we arbitrarily picked the LOD as the changing point for simplicity, which may not happen in practice

For each exposure and a given percent of values below LOD, we pre-determined the LOD values as the corresponding percentile from an independently simulated exposure dataset with sample size 20,000. With each simulated dataset, we first employed each of the five LOD accommodation approaches, then analyzed the resulting datasets with elastic net regression, WQS and BKMR under a unified formulation, that is, $Y=h\left({Z}_{log}\right)+{\alpha }^{T}X+\epsilon ,$ where $h\left({Z}_{log}\right)$ is the exposure–response function. Specifically, $h\left({Z}_{log}\right)$ is ${\beta }^{T}{Z}_{log}$ for elastic net, $\psi ({w}^{T}{\overline{Z} }_{log})$ for WQS with $\psi$ being the total effect of a mixture, $w$ as the vector of weights (or relative importance) and ${\overline{Z} }_{log}$ as the pre-specified quantized ${Z}_{log}$, and a general form $h\left({Z}_{log}\right)$ for BKMR that allows non-linear relationship and interactions (see Appendix A). The R packages ‘glmnet’ [46], ‘gWQS’ [17] and ‘bkmr’ [47] with R version 4.2.1 (The R Foundation for Statistical Computing: http://www.r-project.org/) were used to implement these mixture methods.

In our implementations, all packages in R were applied as a default setting. Tuning parameters for elastic net were obtained from tenfold cross-validation. In WQS, we used quartiles of exposures after applying each approach for handling LOD with 200 bootstrap samples and 60% validation dataset. Five imputed datasets were generated for conventional and truncated MI approaches, and the final estimates of the MI and truncated MI were obtained using Rubin’s rules [44]. The R package ‘bkmrhat’ was used to combine the estimates of the MI and truncated MI in BKMR (https://cran.r-project.org/web/packages/bkmrhat/index.html). We conducted 1000 simulation runs for each scenario. R code is available on GitHub at https://github.com/ml5977/LOD_accommodation.

The goal of our simulation study is to evaluate how different LOD accommodations influence the results of downstream mixture analysis. Note that since we simulated the data, all comparisons are made to estimates from the using the full datasets (i.e., not subject to LOD). We made this choice instead of comparing to the truth because some models are expected to exhibit biases even when all data are observed due to departure from the true underlying model, and certain model coefficients may have different interpretations. For example, elastic net regression explores a bias-variance trade-off, so we expect to see biases due to shrinkage [48]. WQS is based on exposure quantiles, so all the parameters can be interpreted as the average effect when exposures increase by one quantile, whereas the parameter in the true underlying model represents the effect corresponding to a one-unit change. We also do not compare across the three mixture analysis methods, which is beyond the scope of the current study.

For elastic net regression and WQS, we reported the average bias and empirical standard error (SE) of the parameter estimations. For BKMR, using model assessment measures similar to those in Bobb et al. [11], we regressed the estimated exposure–response function $\widehat{h}$ with each LOD accommodation approach on $\widehat{h}$ from the full dataset and reported the average intercept, slope, ${R}^{2}$, and standard error (SE) of $\widehat{h}$ to assess the goodness of fit of the overall effects [11]. An intercept close to 0 and slope close to 1 indicate no influence of the LOD accommodation approach on the downstream mixture analysis. We further reported posterior inclusion probabilities (PIPs) for each exposure. To be consistent with BKMR results in assessing overall effect, ${R}^{2}$ of regressing $\widehat{h}$ from each LOD accommodation on $\widehat{h}$ with the full dataset were also reported for elastic net regression and WQS.

### NHANES data to explore the relationship between POPs and LTL

In addition to the simulation studies, we applied the above LOD accommodation approaches to the NHANES data collected between 2001 and 2002 as described in Gibson et al. [23] and Mitro et al. [32]. We considered a subset of 1,003 participants who were over twenty years old, and provided blood samples and consented to DNA analysis, with sufficient stored samples to estimate telomere length, and without any missing values for individual exposures and covariates not related to LOD, as described in Gibson et al. [23]. The Institutional Review Board of the National Center for Health Statistics approved the survey [49].

To be consistent with Gibson et al. [23], we restricted our analysis to 18 POPs with less than 40% of values below LOD, which include 11 polychlorinated biphenyls (PCBs), 3 dioxins, and 4 furans (Gibson et al. [23]). All samples were measured using high-resolution gas chromatography/isotope-dilution high-resolution mass spectrometry [50, 51]. LODs were typically $\sim 2 ng/g$, although they could be as high as $10.5 ng/g$ [32], and 68.4% of subjects had at least one exposure below LOD. Using the data, Gibson et al. [23] and Mitro et al. [32] hypothesized that exposures to dioxins, furans, and PCBs were associated with longer LTL, which is the outcome of interest in this analysis.

Demographics and exposure levels were described in Gibson et al. [23]. POPs are moderately to highly correlated with Spearman correlation from 0.20 to 0.95 approximately (Gibson et al. [23]). These exposures can be categorized into three groups as described in Gibson et al. [23]: (i) non-dioxin-like PCBs (including PCBs 74, 99, 138, 153, 170, 180, 187 and 194), (ii) non-ortho PCBs (including PCBs 126 and 169), and (iii) all other exposures including mono-ortho-substituted PCB 118, four dibenzo-furans, and three chlorinated dibenzo-p-dioxins, here refered to as mPFDs.

We employed the above-mentioned approaches for accommodating exposures subject to LOD. All exposures were log-transformed due to their right-skewness. We adjusted for all the covariates as in Mitro et al. [32] and Gibson et al. [23], including age, age^2^, sex, race/ethnicity, educational attainment, BMI, serum cotinine, and blood cell count and distribution (white blood cell count, percent lymphocytes, percent monocytes, percent neutrophils, percent eosinophils and percent basophils).

Using the same data, Gibson et al. [23] handled exposure values below LOD through substituting them by LOD/$\sqrt{2}$. They found three potential drivers (PCB 126, PCB 118, and furan 2,3,4,7,8-pncdf) selected by penalized regression methods, a positive overall effect of the POPs by WQS, a positive linear association with furan 2,3,4,7,8-pncdf, suggestive evidence of linear associations with PCBs 126 and 169, and a positive overall effect of the mixture but no interactions among exposures by BKMR. We re-analyzed the data with the same mixture methods after processing the values below LOD with five LOD accommodation approaches.

We recognized the need for sampling weights to account for the complex NHANES sampling scheme, in order to obtain results generalizable to the US population [49]. However, our goal was to empirically compare the impact of different LOD accommodation approaches, rather than to provide estimates generalizable to the population. Thus, we simplified our analysis here by not including sampling weights for the NHANES cohort, so our results were consistent with those in Gibson et al. [23]. We do recommend incorporating sampling weights into the analysis if an generalizable estimate is needed. We note some of the mixture analysis methods explored here, such as BKMR and WQS, require additional efforts to appropriately incorporate sampling weights, which is beyond the scope of this paper.

## Results

### Simulation results: elastic net regression

Depending on the scenarios, the overall percent of subjects without any value below LOD in the simulated data was approximately 30% to 40%. Table 1 showed the bias of exposures ${Z}_{1}$ to ${Z}_{3}$ (group 1) and ${R}^{2}$ for each LOD accommodation approach with elastic net regression, while all other results for elastic net is in Table S1.

**Table 1:** Bias (SE) for exposures in Group 1 and ${R}^{2}$ with elastic net regression and each LOD accommodation approach compared to using full dataset

LOD accommodation	Moderate correlation ($\boldsymbol\sigma\boldsymbol=\mathbf1\boldsymbol/\mathbf2$)				High correlation ($\boldsymbol\sigma\boldsymbol=\mathbf1\boldsymbol/\mathbf8$)			
	* β*_1_	* β*_2_	*β*_*3*_	*R*^*2*^	*β*_*1*_	*β*_*2*_	*β*_*3*_	*R*^*2*^
Scenario 1								
Complete case	-0.12 (0.26)	-0.13 (0.28)	0.02 (0.21)	0.74	-0.16 (0.65)	-0.12 (0.41)	-0.03 (0.42)	0.60
LOD/$\sqrt{2}$	0.03 (0.14)	-0.03 (0.14)	0.01 (0.11)	0.84	-0.03 (0.53)	-0.16 (0.23)	-0.04 (0.22)	0.79
MI	0.05 (0.14)	0.02 (0.20)	0.02 (0.20)	0.80	0.06 (0.52)	0.12 (0.50)	-0.03 (0.53)	0.82
Truncated MI	0.00 (0.14)	0.01 (0.15)	0.00 (0.12)	0.83	0.04 (0.52)	0.08 (0.46)	-0.01 (0.40)	0.85
F-AFT	0.02 (0.14)	0.01 (0.15)	0.00 (0.12)	0.84	0.02 (0.53)	-0.06 (0.41)	-0.01 (0.34)	0.84
Scenario 2A								
Complete case	0.00 (0.26)		0.09 (0.22)	0.52	-0.17 (0.65)		0.00 (0.40)	0.56
LOD/$\sqrt{2}$	0.14 (0.14)		0.10 (0.13)	0.62	-0.01 (0.54)		-0.02 (0.21)	0.77
MI	0.15 (0.15)		0.12 (0.20)	0.61	0.09 (0.55)		0.05 (0.50)	0.77
Truncated MI	0.15 (0.15)		0.13 (0.20)	0.61	0.07 (0.54)		0.04 (0.48)	0.78
F-AFT	0.14 (0.14)		0.11 (0.14)	0.62	0.03 (0.54)		0.01 (0.32)	0.80
Scenario 2B								
Complete case	-0.04 (0.24)	-0.26 (0.26)	0.06 (0.21)	0.64	-0.19 (0.63)	-0.27 (0.14)	-0.01 (0.39)	0.54
LOD/$\sqrt{2}$	0.09 (0.14)	-0.18 (0.17)	0.06 (0.12)	0.73	-0.05 (0.54)	-0.26 (0.12)	-0.02 (0.20)	0.76
MI	0.10 (0.14)	-0.17 (0.17)	0.06 (0.20)	0.73	0.05 (0.54)	-0.25 (0.14)	0.03 (0.48)	0.76
Truncated MI	0.09 (0.14)	-0.17 (0.17)	0.07 (0.19)	0.73	0.03 (0.54)	-0.26 (0.13)	0.02 (0.46)	0.78
F-AFT	0.09 (0.14)	-0.18 (0.17)	0.06 (0.13)	0.74	-0.01 (0.54)	-0.26 (0.13)	0.01 (0.31)	0.80
Scenario 3								
Complete case	-0.10 (0.26)	-0.28 (0.30)	0.01 (0.21)	0.71	-0.17 (0.35)	-0.34 (0.35)	0.02 (0.26)	0.65
LOD/$\sqrt{2}$	0.00 (0.14)	0.13 (0.15)	-0.01 (0.11)	0.85	0.00 (0.14)	0.16 (0.15)	-0.01 (0.11)	0.86
MI	0.07 (0.15)	-0.03 (0.22)	0.03 (0.20)	0.78	0.07 (0.15)	0.00 (0.25)	0.04 (0.24)	0.77
Truncated MI	-0.03 (0.14)	0.18 (0.15)	-0.02 (0.12)	0.83	-0.04 (0.14)	0.22 (0.16)	-0.03 (0.12)	0.83
F-AFT	0.00 (0.14)	0.15 (0.15)	-0.02 (0.12)	0.84	0.00 (0.14)	0.19 (0.16)	-0.03 (0.12)	0.84
Scenario 4								
Complete case	-0.12 (0.26)	-0.10 (0.30)	0.02 (0.22)	0.74	-0.19 (0.32)	-0.18 (0.35)	0.03 (0.28)	0.67
LOD/$\sqrt{2}$	0.00 (0.14)	0.10 (0.15)	0.00 (0.11)	0.86	0.00 (0.13)	0.14 (0.15)	0.00 (0.11)	0.86
MI	0.04 (0.14)	0.06 (0.22)	0.03 (0.18)	0.81	0.05 (0.14)	0.06 (0.23)	0.03 (0.22)	0.79
Truncated MI	-0.02 (0.14)	0.12 (0.15)	-0.01 (0.11)	0.84	-0.02 (0.13)	0.15 (0.15)	-0.02 (0.12)	0.84
F-AFT	0.00 (0.14)	0.12 (0.16)	-0.01 (0.12)	0.85	0.00 (0.13)	0.15 (0.16)	-0.02 (0.12)	0.85

Bias (SE) was reported for exposures in Group 1 (${\beta }_{1},{\beta }_{2}$ and ${\beta }_{3}$). All other results are provided in Table S1. All comparisons were made to the parameters with full datasets without LOD. ${R}^{2}$ was calculated by regression $\widehat{h}$ from each LOD accommodation on $\widehat{h}$ with the full dataset. In Scenario 2A, ${\beta }_{2}$ was not estimated because ${Z}_{2}$ was not included in the analysis

*Abbreviations:* Imputation by LOD/$\sqrt{2}$ (LOD/$\sqrt{2}$), *MI* Conventional multiple imputation, *Truncated MI* Truncated multiple imputation, *F-AFT* Imputation by estimates using the AFT model

In Scenario 1 as a general case, when the exposures were moderately correlated, most approaches were unbiased except for the complete case analysis which also had higher SE, indicating inefficiency. In the high correlation setting, the biases in complete case analysis persisted, while imputing values below LOD by LOD/$\sqrt{2}$ and conventional MI also showed biases for ${\beta }_{2}$. The bias in MI decreased when truncated MI was used. Imputation by estimates from the AFT model (F-AFT) and truncated MI were empirically unbiased and efficient in both moderate and high correlation settings under Scenario 1.

When ${Z}_{2}$ was subject to 70% values below LOD and was completely ignored in the elastic net regression (Scenario 2A), all LOD accommodations performed poorly with low ${R}^{2}$ and large biases for exposures in the same group (${\beta }_{1} {\text{and}} {\beta }_{3}$) and covariate ${X}_{1}$ (${\alpha }_{1}$). Note that exposures in other groups were relatively less impacted since the effect of ${Z}_{2}$ was potentially accounted for by those in the same group (i.e., ${Z}_{1}$ and ${Z}_{3}$). These biases decreased when correlations were higher, again presumably because the information in ${Z}_{2}$ was better captured by other exposures in the same group. These biases in ${\beta }_{1}$ and ${\beta }_{3}$ were further alleviated when an indicator variable of ${Z}_{2}$ (Scenario 2B), i.e., $I({Z}_{2}>{\text{LOD}}$), was used. However, ${\beta }_{2}$ now has a different interpretation (i.e., the difference between values above LOD versus below the LOD), so we expected to see its large bias. Although Group 1 exposures were still biased in Scenario 2B for most of the LOD accommodation approaches, F-AFT and truncated MI generally performed well, especially in high correlation setting, followed by imputation by LOD/$\sqrt{2}$ and conventional MI.

When different distributions below and above LOD were assumed for ${Z}_{2}$ (Scenario 3) or ${Z}_{2}$ had different effects below and above LOD (Scenario 4), all approaches for handling LOD, including model-based approaches such as truncated MI and F-AFT, performed poorly for ${\beta }_{2}$ because we lack any information to make inference about the values and relationship below LOD. Surprisingly, we observed a smaller bias of ${\beta }_{2}$ with conventional MI. However, the bias increased dramatically when the percent of values below LOD increased (results not shown). Furthermore, conventional MI was substantially biased in the intercept ${\alpha }_{0}$ and was inefficient in ${\beta }_{2}$ compared to other LOD approaches with a lower ${R}^{2}.$ Therefore, truncated MI and F-AFT still performed relatively better than other approaches and using LOD/$\sqrt{2}$ yielded slightly worse results but comparable.

### Simulation results: WQS regression

We summarized the ${\beta }_{1}$ to ${\beta }_{3}$ and ${R}^{2}$ results in Table 2 and all remaining results for WQS in Table S2. We expected WQS to be less sensitive to values below LOD due to using quantized exposures. However, some LOD accommodations could disrupt the quantiles and result in large biases. For example, in Scenario 1, we found that F-AFT and truncated MI mostly maintained the exposures’ quantiles and were empirically unbiased and efficient (Table 2). Complete case analysis showed relatively large biases, especially for overall effect estimate ($\psi$) in the setting of moderate correlation, due to the loss of all values below LOD and complete change of quantiles. Conventional MI also showed slightly larger biases compared to truncated MI because the imputed values could occasionally exceed the detection limit that can change quantile estimates. When LOD/$\sqrt{2}$ was used, performance was unstable because the exposure’s quantiles may not be maintained in the analysis of WQS if the percent of value below LOD is high (e.g., potential mis-assignment of quantiles). In evaluating the overall effects of the mixture with ${R}^{2}$, complete case analysis underperformed across all LOD approaches while the others were similar.

**Table 2:** Bias (SE) for exposures in Group 1 and ${R}^{2}$ with WQS and each LOD accommodation approach compared to using full dataset

LOD accommodation	Moderate correlation ($\boldsymbol\sigma\boldsymbol=\mathbf1\boldsymbol/\mathbf2$)					High correlation ($\boldsymbol\sigma\boldsymbol=\mathbf1\boldsymbol/\mathbf8$)				
	$\boldsymbol\psi$	${\boldsymbol w}_{\mathbf1}$	${\boldsymbol w}_{\mathbf2}$	${\boldsymbol w}_{\mathbf3}$	$\boldsymbol R^{\mathbf2}$	$\boldsymbol\psi$	${\boldsymbol w}_{\mathbf1}$	${\boldsymbol w}_{\mathbf2}$	${\boldsymbol w}_{\mathbf3}$	$\boldsymbol R^{\mathbf2}$
Scenario 1										
Complete case	-0.34 (0.33)	-0.03 (0.10)	-0.04 (0.10)	0.02 (0.06)	0.73	-0.03 (0.33)	-0.02 (0.13)	0.01 (0.13)	0.01 (0.10)	0.87
LOD/$\sqrt{2}$	0.15 (0.22)	-0.02 (0.06)	0.00 (0.08)	0.01 (0.05)	0.86	0.01 (0.26)	0.00 (0.13)	-0.02 (0.11)	0.00 (0.10)	0.92
MI	-0.06 (0.18)	0.03 (0.06)	-0.05 (0.06)	0.00 (0.04)	0.79	0.01 (0.24)	0.00 (0.13)	0.00 (0.11)	0.00 (0.09)	0.93
Truncated MI	0.00 (0.18)	0.00 (0.06)	0.00 (0.07)	0.00 (0.04)	0.84	0.01 (0.24)	0.00 (0.13)	0.00 (0.11)	0.00 (0.10)	0.93
F-AFT	-0.01 (0.18)	0.00 (0.06)	0.00 (0.07)	0.00 (0.04)	0.85	-0.01 (0.24)	0.00 (0.13)	-0.01 (0.11)	0.00 (0.10)	0.93
Scenario 2A										
Complete case	-0.49 (0.29)	0.08 (0.12)		0.05 (0.08)	0.54	-0.05 (0.30)	0.02 (0.14)		0.03 (0.11)	0.83
LOD/$\sqrt{2}$	-0.17 (0.19)	0.08 (0.07)		0.05 (0.07)	0.65	-0.01 (0.25)	0.03 (0.14)		0.02 (0.11)	0.88
MI	-0.28 (0.17)	0.11 (0.07)		0.02 (0.05)	0.63	-0.01 (0.23)	0.03 (0.14)		0.02 (0.10)	0.90
Truncated MI	-0.28 (0.17)	0.12 (0.07)		0.03 (0.05)	0.63	-0.01 (0.23)	0.04 (0.14)		0.02 (0.11)	0.90
F-AFT	-0.28 (0.17)	0.11 (0.07)		0.04 (0.06)	0.65	-0.02 (0.23)	0.04 (0.14)		0.02 (0.12)	0.90
Scenario 2B										
Complete case	-0.33 (0.34)	-0.14 (0.04)	-0.20 (0.05)	0.04 (0.07)	0.45	-0.01 (0.34)	-0.06 (0.04)	-0.06 (0.08)	0.01 (0.10)	0.82
LOD/$\sqrt{2}$	0.09 (0.22)	-0.11 (0.04)	-0.22 (0.03)	0.04 (0.06)	0.57	0.04 (0.29)	-0.06 (0.05)	-0.07 (0.07)	0.01 (0.11)	0.88
MI	-0.10 (0.19)	-0.15 (0.02)	-0.21 (0.03)	0.01 (0.04)	0.50	0.03 (0.25)	-0.08 (0.01)	-0.07 (0.07)	0.01 (0.09)	0.89
Truncated MI	-0.12 (0.19)	-0.15 (0.02)	-0.21 (0.03)	0.02 (0.05)	0.50	0.03 (0.26)	-0.08 (0.01)	-0.07 (0.07)	0.01 (0.10)	0.89
F-AFT	-0.12 (0.19)	-0.16 (0.02)	-0.21 (0.03)	0.03 (0.05)	0.52	0.01 (0.26)	-0.08 (0.01)	-0.07 (0.08)	0.01 (0.10)	0.89
Scenario 3										
Complete case	-0.46 (0.35)	0.00 (0.10)	-0.11 (0.10)	0.03 (0.06)	0.69	-0.54 (0.44)	-0.03 (0.11)	-0.12 (0.11)	0.04 (0.07)	0.66
LOD/$\sqrt{2}$	0.16 (0.23)	-0.01 (0.06)	-0.01 (0.08)	0.01 (0.04)	0.85	0.20 (0.24)	-0.02 (0.06)	0.01 (0.07)	0.01 (0.04)	0.86
MI	-0.14 (0.20)	0.05 (0.07)	-0.10 (0.07)	0.01 (0.04)	0.75	-0.14 (0.21)	0.05 (0.07)	-0.10 (0.07)	0.01 (0.04)	0.74
Truncated MI	0.03 (0.19)	-0.01 (0.06)	0.01 (0.07)	0.00 (0.04)	0.83	0.05 (0.19)	-0.01 (0.06)	0.02 (0.07)	0.00 (0.04)	0.83
F-AFT	0.01 (0.20)	0.00 (0.06)	0.00 (0.07)	0.00 (0.04)	0.84	0.01 (0.20)	0.00 (0.06)	0.00 (0.07)	0.00 (0.04)	0.84
Scenario 4										
Complete case	-0.35 (0.34)	-0.02 (0.11)	-0.06 (0.10)	0.03 (0.06)	0.72	-0.45 (0.42)	-0.05 (0.11)	-0.08 (0.10)	0.04 (0.07)	0.69
LOD/$\sqrt{2}$	0.18 (0.22)	-0.02 (0.06)	0.01 (0.08)	0.01 (0.04)	0.86	0.20 (0.22)	-0.03 (0.06)	0.02 (0.08)	0.01 (0.04)	0.87
MI	-0.08 (0.18)	0.03 (0.07)	-0.07 (0.07)	0.00 (0.04)	0.78	-0.08 (0.18)	0.04 (0.07)	-0.07 (0.06)	0.00 (0.04)	0.76
Truncated MI	0.02 (0.17)	0.00 (0.07)	0.01 (0.07)	0.00 (0.04)	0.84	0.03 (0.18)	-0.01 (0.06)	0.02 (0.07)	0.00 (0.04)	0.84
F-AFT	0.00 (0.18)	0.00 (0.07)	0.00 (0.07)	0.00 (0.04)	0.85	0.00 (0.18)	0.00 (0.07)	0.00 (0.07)	0.00 (0.04)	0.85

Bias (SE) was reported for the total effect ($\psi$) and exposures in group 1 (${w}_{1},{w}_{2}$ and ${w}_{3}$). All other results are provided in Table S2. All comparisons were made to the parameters with full datasets without LOD. ${R}^{2}$ was calculated by regression $\widehat{h}$ from each LOD accommodation on $\widehat{h}$ with the full dataset. In Scenario 2A, ${w}_{2}$ was not estimated because ${Z}_{2}$ was not included in the analysis

*Abbreviations:* Imputation by LOD/$\sqrt{2}$ (LOD/$\sqrt{2}$), *MI* Conventional multiple imputation, *Truncated MI* Truncated multiple imputation, *F-AFT* Imputation by estimates using the AFT model

When the percent of values below the LOD for ${Z}_{2}$ was increased to 70% and ${Z}_{2}$ was ignored in the WQS analysis (Scenario 2A), in the moderate correlation setting, the biases increased, especially for effects of exposures in the same group (${Z}_{1}$ and ${Z}_{3}$), total effect $\psi$, intercept and covariate ${X}_{1}$. When an indicator variable $I({Z}_{2}>LOD)$ was used as in Scenario 2B, the bias of total effects was slightly alleviated, but biases in weights of group 1 exposures, intercept and covariate ${X}_{1}$ persisted. All LOD accommodations performed similarly well in the high correlation setting, except complete case analysis was substantially biased in intercept and with lower ${R}^{2}$. In the scenarios with different distributions (Scenario 3) or different effects (Scenario 4) below and above LOD for ${Z}_{2}$, truncated MI and F-AFT maintained better performance in both parameter estimates and ${R}^{2}$ compared to the other LOD accommodation approaches. Imputation by LOD/$\sqrt{2}$ showed similar ${R}^{2}$, but there was a large bias in estimating the total effect $\psi$.

### Simulation results: BKMR

Table 3 showed the simulation results of BKMR under different scenarios. In Scenario 1, F-AFT performed the best among all the approaches, with intercept close to 0 and slope close to 1, indicating empirically unbiased results of $h\left({Z}_{log}\right)$. The F-AFT also led to high ${R}^{2}$ and lower SE. Truncated MI performed similarly to F-AFT but was slightly less efficient. Complete case analysis and imputation by LOD/$\sqrt{2}$ underperformed, especially in the high correlation setting. In Scenario 2, none of the LOD accommodation approaches performed satisfactorily, despite the indicator variable (Scenario 2B) resulting in slightly better estimation than Scenario 2A. In Scenarios 3 and 4, F-AFT and truncated MI were the most unbiased and efficient in both correlation settings. In identifying important mixture components by PIPs, F-AFT and truncated MI performed similarly to using the full dataset, while complete case analysis showed discrepancies (Figure S2). The performance of imputation by LOD/$\sqrt{2}$ in PIPs was comparable to those of F-AFT and truncated MI, despite this approach showing unstable results in some cases (e.g., high correlation settings).

**Table 3:** Summary measures of estimated $h\left(Z\right)$with BKMR and each LOD accommodation approach compared to using full dataset

LOD accommodation	Moderate correlation ($\boldsymbol\sigma\boldsymbol=\mathbf1\boldsymbol/\mathbf2$)				High correlation ($\boldsymbol\sigma\boldsymbol=\mathbf1\boldsymbol/\mathbf8$)			
	Intercept	Slope	$\boldsymbol R^{\mathbf2}$	SE	Intercept	Slope	$\boldsymbol R^{\mathbf2}$	SE
Scenario 1								
Complete case	0.07	0.91	0.83	0.42	0.58	0.68	0.56	0.21
LOD/$\sqrt{2}$	0.11	0.94	0.96	0.28	0.44	0.77	0.75	0.16
MI	0.23	0.88	0.87	0.30	-0.23	1.14	0.81	0.18
Truncated MI	0.05	0.98	0.95	0.32	-0.21	1.13	0.85	0.18
F-AFT	0.08	0.97	0.96	0.28	0.12	0.95	0.82	0.16
Scenario 2A								
Complete case	0.54	0.68	0.70	0.37	0.79	0.57	0.51	0.20
LOD/$\sqrt{2}$	0.47	0.72	0.82	0.25	0.50	0.74	0.73	0.16
MI	0.47	0.72	0.79	0.27	0.23	0.89	0.73	0.17
Truncated MI	0.45	0.72	0.81	0.27	0.28	0.86	0.75	0.17
F-AFT	0.47	0.72	0.82	0.25	0.39	0.80	0.77	0.15
Scenario 2B								
Complete case	0.31	0.79	0.78	0.40	0.80	0.55	0.48	0.21
LOD/$\sqrt{2}$	0.31	0.81	0.89	0.27	0.55	0.71	0.71	0.16
MI	0.31	0.82	0.87	0.29	0.23	0.88	0.72	0.18
Truncated MI	0.29	0.82	0.88	0.29	0.29	0.85	0.74	0.18
F-AFT	0.31	0.82	0.89	0.27	0.45	0.77	0.75	0.16
Scenario 3								
Complete case	0.29	0.87	0.81	0.43	0.36	0.80	0.70	0.50
LOD/$\sqrt{2}$	0.05	0.98	0.95	0.31	0.07	0.98	0.95	0.30
MI	0.30	0.84	0.83	0.33	0.33	0.83	0.81	0.33
Truncated MI	0.00	1.03	0.92	0.36	0.01	1.04	0.91	0.36
F-AFT	0.04	1.01	0.95	0.31	0.06	1.01	0.95	0.31
Scenario 4								
Complete case	0.18	0.89	0.82	0.43	0.24	0.82	0.72	0.50
LOD/$\sqrt{2}$	0.05	0.98	0.96	0.28	0.05	0.98	0.96	0.28
MI	0.21	0.89	0.88	0.30	0.24	0.88	0.86	0.30
Truncated MI	-0.01	1.02	0.95	0.32	-0.01	1.02	0.94	0.32
F-AFT	0.03	1.01	0.97	0.28	0.03	1.01	0.96	0.28

Summary measures were obtained by regressing the estimated$\widehat{h}$of each LOD-accommodation approach on$\widehat{h}$using full datasets, and reported average intercept, slope, and${R}^{2}$across simulation iterations. Zero intercept and 1 of slope indicate LOD accommodation approach does not influence BKMR results. “SE” denotes the posterior standard deviation of the$\widehat{h}$

*Abbreviations:* Imputation by LOD/$\sqrt{2}$(LOD/$\sqrt{2}$), *MI* Conventional multiple imputation, *Truncated MI* Truncated multiple imputation, *F-AFT* Imputation by estimates using the AFT model

### NHANES data analysis results

**Fig. 1:**
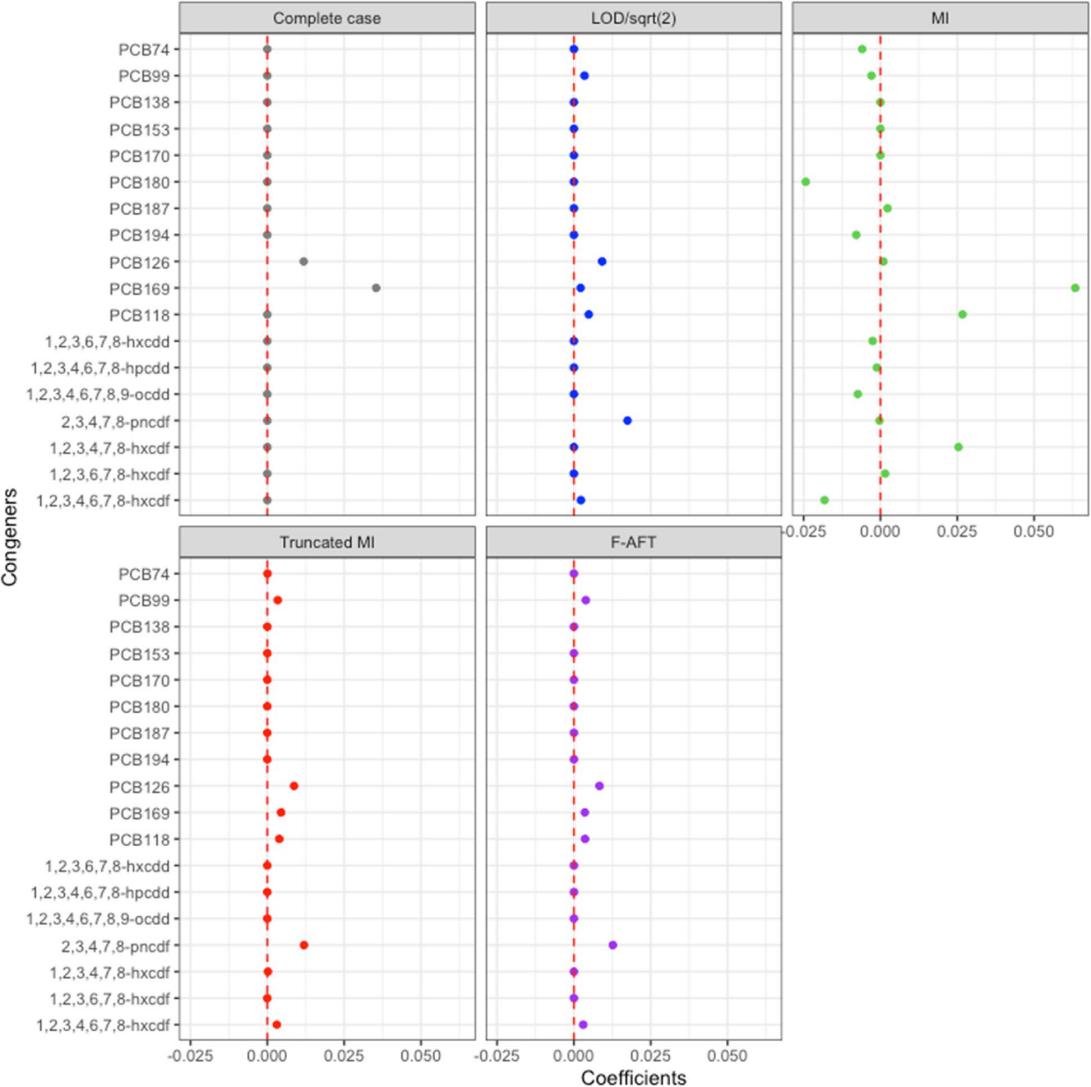
Coefficients for 18 POPs with elastic net regression and each LOD approach using NHANES data 2001–2002. Abbreviations: Imputation by LOD/$\sqrt{2}$ (LOD/sqrt(2)); conventional multiple imputation (MI); truncated multiple imputation (Truncated MI); imputation by estimates using the AFT model (F-AFT)

When applying the elastic net regression to the mixture, F-AFT, truncated MI, and imputation by LOD/$\sqrt{2}$ generally resulted in similar findings (Fig. 1). Specifically, they all identified six important POPs: PCB99, PCB118, PCB126, PCB169, furan 2,3,4,7,8-pncdf, and furan 1,2,3,4,6,7-hxcdf with similar effects. Complete case analysis only identified PCB126 and PCB169 to be important, while conventional MI resulted in selecting many more exposures. We additionally conducted group lasso with the 18 POPs categorized into three groups: non-dioxin-like PCBs, non-ortho-PCBs, and mPFD, as described above. None of exposures in non-dioxin-like PCBs were selected except when using conventional MI, while non-ortho PCBs (i.e., PCB126 and PCB169) were associated with non-zero coefficients in all LOD approaches (Figure S3). The magnitudes of the non-ortho PCB effects were larger with complete case analysis and conventional MI while the other three approaches yielded similar effects. For the mPFD exposures, again, F-AFT, truncated MI and imputaiton by LOD/$\sqrt{2}$ estimated similar coefficients and they all selected furan 2,3,4,7,8-pncdf as the most important exposures, followed by PCB118. Complete case analysis resulted in null effects for all mPFDs, and conventional MI showed mild effects for some non-dioxin-like PCBs and opposite direction for some of the mPFDs.

Deciles in exposures were used in the analysis of WQS regression to be consistent with Gibson et al. [23]. The total effect of 18 POPs ranged between 0.014 and 0.018 under various LOD-handling approaches, and they were statistically significant except for with complete case analysis, which is expected due to loss of efficiency with only 317 subjects included in the analysis (Table 4). Applying a priori cut-off weight of 1/18, we found 3 to 4 important POPs across these LOD accommodation approaches. Imputation by LOD/$\sqrt{2}$ and truncated MI found 2,3,4,7,8-pncdf as the most important exposure, followed by PCB126 and 1,2,3,4,6,7,8-hxcdf. In addition to these three, the F-AFT approach also identified PCB194.

**Table 4:** Total effect of 18 POPs and important exposures identified from WQS with each LOD accommodation approach, with NHANES 2001–2002

	Total effect $\boldsymbol\psi$ (SE)	Important exposures (weight)
Complete case	0.014 (0.009)	PCB169 (0.538), 1,2,3,4,6,7,8-hxcdf (0.125), PCB126 (0.113)
LOD/$\sqrt{2}$	0.018 (0.005)	2,3,4,7,8-pncdf (0.350), PCB126 (0.186), 1,2,3,4,6,7,8-hxcdf (0.184)
MI	0.015 (0.006)	PCB169 (0.580), 1,2,3,4,6,7,8-hxcdf (0.153), PCB126 (0.080)
Truncated MI	0.018 (0.005)	2,3,4,7,8-pncdf (0.346), PCB126 (0.186), 1,2,3,4,6,7,8-hxcdf (0.183)
F-AFT	0.017 (0.005)	2,3,4,7,8-pncdf (0.328), PCB126 (0.189), 1,2,3,4,6,7,8-hxcdf (0.184), PCB194 (0.062)

Relative importance was reported for exposures with the weight over 1/18

*Abbreviations:* Imputation by LOD/$\sqrt{2}$ (LOD/$\sqrt{2}$), *MI* Conventional multiple imputation, *Truncated MI* Truncated multiple imputation, *F-AFT* Imputation by estimates using the AFT model

**Fig. 2:**
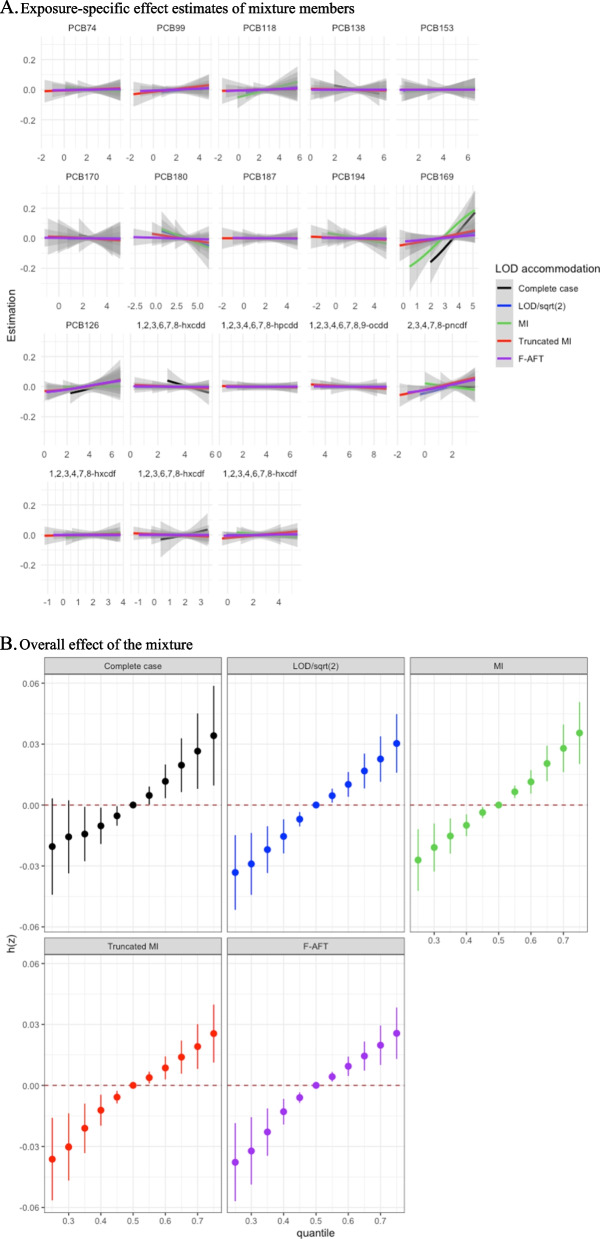
Individual and overall relationships of 18 POPs with log-LTL from BKMR using NHANES 2001–2002 data. **A** Exposure-specific effect estimates of mixture members. **B** Overall effect of the mixture. Abbreviations: Imputation by LOD/$\sqrt{2}$ (LOD/sqrt(2)); conventional multiple imputation (MI); truncated multiple imputation (Truncated MI); imputation by estimates using the AFT model (F-AFT)

Using BKMR, we employed hierarchical variable selection with the three pre-defined groups, which provided importance scores for both the groups (i.e., group PIPs) and each exposure within a group (i.e., conditional PIPs). Truncated MI and imputation by LOD/$\sqrt{2}$ both resulted in the non-ortho PCB group with the highest PIP among three groups, while mPFD group has the highest PIP with F-AFT, conventional MI and complete case analysis (Table S3). Within the mPFD exposures, furan 2,3,4,7,8-pncdf contributed most to the model, followed by PCB 118 when imputation by LOD/$\sqrt{2}$, truncated MI and F-AFT approaches were used. PCB 169 and PCB 126 in the non-ortho PCB group had similar importance weights when we applied imputation by LOD/$\sqrt{2}$, truncated MI and F-AFT approaches. The individual effects of the POP exposures showed linear trends across LOD accommodation approaches (Fig. 2A), while the magnitudes of associations varied, especially for PCB169 and furan 2,3,4,7,8-pncdf which were selected as important exposures among the 18 POPs. The overall mixture effect was also close to a positive linear trend on the LTL outcome across LOD approaches, while the strength and efficiency varied (Fig. 2B).

## Discussion

In this study we have compared how five popular approaches for handling exposures subject to LOD influence the results of mixture analysis. We did not mean to provide a guideline on how to handle values below LOD, rather to draw attention about how results can be misled by the various LOD accommodation approaches, and would like to advocate for careful examination of LOD accommodation prior to applying downstream mixtures analysis.

Through our extensive simulations, we generally favored using truncated MI and censored AFT models to impute values below LOD for the stability of downstream mixture analysis when the percent of the LOD was low to moderate (e.g., 30–50%). Compared to other approaches, truncated MI and censored AFT models generate imputed values based on the information from other exposures and covariates and guarantee that the imputed values are below LOD. Satisfactory results were also found with these two approaches when evaluating statistical uncertainties, such as mean squared error and coverage probability of the 95% confidence interval, in additional linear regression simulations (Table S4), as well as when incorporating grouping information in the analysis of group lasso and BKMR with hierarchical variable selection (Tables S5 and S6). Of course, these model-based approaches rely on modeling assumptions and borrowing information from other exposures and baseline demographics. However, we argue since we do not get to observe any information below LOD, we need some assumptions, and the modeling assumptions made in these two approaches are relatively mild and reasonable in practice.

Complete case analysis and imputations by LOD/$\sqrt{2}$ are frequently used in environmental health studies due to their easy implementation. However, we found that their performance can be quite unstable, especially in scenarios with high correlations or high percent of values below the LOD as commonly observed in environmental mixture studies. Richardson and Ciampi [38] also reported the bias in risk estimates when an arbitrary constant value such as LOD or LOD/2 was used to replace values below the LOD, and pointed out the magnitude of bias depends on the differences between the substitution value and true exposure distribution below LOD.

When the percent of values below LOD increased to 70% in our simulations, using an indicator variable of whether the values are above LOD performed better than excluding the exposure variable in the analysis. When other exposures were highly correlated with the exposure that had a high percent of values below LOD, its influence on the overall effect was limited because its information was well captured by other exposures. Based on our simulation studies with various percent of values below the LOD (results not shown), we recommend using the indicator variable approach when the percent of exposures below LOD is above 50%. For the NHANES data analysis, we restricted to exposures with less than 40% of values below LOD, in order to replicate the analysis in Gibson et al. [23]. If we were to perform our own analysis, we will likely use 50% as a cutoff to include three additional POPs in the analysis.

We acknowledge that it is difficult to verify an assumed relationship or distribution between exposure subject to LOD and disease outcome for values below the LOD. To address this, we examined various LOD accommodation approaches assuming that the relationship has no impact below the LOD (Scenario 3) and the distribution is different for values below LOD (Scenario 4) as a case study. In our simulation study, none of the approaches for handling LOD in this study performed satisfactorily, which is similar to the results given by Ortega-Villa et al. [33] for a single exposure. In such cases, we recommend considering the binary indicator approach for exposures with suspected differential distribution or relationship with outcome [52], while truncated MI or F-AFT can still be used for all other exposures subject to LOD. Even though BKMR with imputation by LOD/$\sqrt{2}$, truncated MI and F-AFT performed satisfactorily in such scenarios due to its flexibility in allowing non-linear relationship, the implementation of the missing indicator approach could lead to further performance enhancement in BKMR. Yet, interpreting the estimated coefficient for the missing indicator within the exposure–response function of BKMR might prove challenging, especially when indicators are needed for multiple exposures.

We applied the LOD approaches to NHANES 2001–2002 where 18 selected POPs were subject to different proportions of values below the LOD. In our analysis, we did not include sampling weights because our goal was to understand the impact of different LOD accommodation approaches on downstream mixture analysis as a comparison with Gibson et al. [23], which did not incorporate sampling weights. To incorporate sampling weights, Zhang et al. [53] sampled one bootstrap sample with replacement from the NHANES data, with probabilities proportional to the sampling weights to test the results. We also implemented the same procedure. Although the mixture analysis results were different, we observed similar patterns across LOD accommodation approaches (results not shown).

In this study, we considered a two-stage approach as a practical implementation where we first performed the LOD accommodation to get a “complete” dataset, then conducted mixture analyses using this dataset. In the multiple imputation (MI) with or without truncation, we generated five imputed datasets, and combined the results of mixture analysis using the Rubin’s rule [44], which takes imputation variability into account in the final results. However, single imputations by LOD/$\sqrt{2}$, and by estimates from the censored AFT model did not account for the uncertainty resulting from the imputation, which could lead to an overestimation of the precision. This can also be seen in the linear regression simulation results in Table S4, with somewhat worse coverage probabilities by F-AFT and LOD/$\sqrt{2}$. Nevertheless, in our experience working with epidemiologists, this two-stage approach is highly preferred in practice due to its convenience. It requires handling the LOD only once and allows the resulting dataset to be used as the “true” dataset for multiple studies in the future. As mentioned above, Chen et al. [41] proposed a semiparametric multivariate AFT approach with multiple exposures to simultaneously model the exposures subject to LOD and the outcome, which accounts for uncertainty in the exposure assessment. This approach was applied to study the relationship between a panel of urinary trace metals and oxidative stress in pregnant women. The use of this powerful approach is limited by its computational complexity. Thus, it is of great interest to extend this approach to allow simultaneous modeling of the exposures subject to LOD with various mixture outcome models, and provide user-friendly software.

Some analytical laboratories often provide the machine readings for specimens whose observed values is declared to be below the LOD, with the understanding that the specimen’s level of analyte cannot reliably distinguished from zero; these readings may involve substantial measurements errors. Machine-read values have been often used in environmental mixture studies [54–56]. However, we did not consider the machine-read approach in our case study because it is difficult to justify the actual mechanism of the machine-raed approach given that each machine in each lab has its unique way of generating the reads, and the accuracy could vary dramatically. In our data analysis, NHANES 2001–2002 also did not provide machine-read values.

Here, we limited to three mixture analysis methods including elastic net regression, WQS, and BKMR which have been widely used in environmental mixture studies. We are aware of many other mixtures anslysis methods and performed simulations to understand the impact of LOD accommodations on these methods too. However, they were not included due to the length of the current manuscript. For example, Keil et al. [57] recently proposed a quantile-based g-computation method (q-gcomp) that builds up on WQS regression integrating its estimation procedure with a g-computation technique, which is widely used for causal inference [58]. The q-gcomp method relaxes the unidirectionality and linearity assumptions of the WQS regression. Results were similar to those for WQS, which is likely due to their similar model structures and our simulated exposures were all in one direction (e.g., see Table S7 for q-gcomp results under Scenario 1).

Several methodological extensions are of interest for further exploration. First, in this study we assumed a linear combination of variables in MI and F-AFT for imputation. However, these approaches could allow non-linearity and/or non-additivity for better recovering the true effects in the mixture setting. We also assumed all the effects were in the same direction with no interactions, which limits generalizability, and assumed a multivariate normal distribution for the exposures. Second, our study employed popular approaches for accommodating LOD before applying the mixture analysis methods to the revised data (i.e., a two-stage approach). Lastly, environmental mixture exposures are often repeatedly measured (i.e., longitudinal mixture exposures), which could allow more accurate modeling of the exposure trajectories. We leave a consideration of LOD adjustments that can appropriately incorporate longitudinal mixture exposures as a project for further development.

## Conclusion

Quantifying the impact of mixtures of environmental exposures is becoming increasingly important for identifying disease risk factors and developing targeted public health interventions. Our case study delved into the issue of LOD in detail to understand how common approaches for handling LOD impact downstream mixture analysis. Our exploration provides insight into various LOD accommodation approaches in downstream mixture analyses, enhancing the quality and reliability of environmental health studies.

### Supplementary Information

Supplementary Material 1.

## Data Availability

The data that support the findings in this paper are available on GitHub at https://github.com/lizzyagibson/SHARP.Mixtures.Workshop, published along side Gibson et al. (2019) [23]. R code for LOD accommodation approaches is available at https://github.com/ml5977/LOD_accommodation.

## Appendix A. Detailed descriptions of mixture analysis methods

For each subject $i \left(i=1,\dots ,n\right)$, let ${Y}_{i}$ be a continuous outcome of interest. Let ${Z}_{i}$ and ${X}_{i}$ denote $p$- and $q$-vector of exposures and covariates, respectively. Note that ${X}_{i}$ includes 1 for the intercept term. Thus, we observe data $\left\{{Y}_{i}, {Z}_{i}, {X}_{i}, i=1,\dots ,n\right\}$ with sample size $n$. Using these notations, ordinary linear regression can be specified as ${Y}_{i}={\beta }^{T}{Z}_{i}+{\alpha }^{T}{X}_{i}+{\epsilon }_{i}$, with ${\epsilon }_{i} \sim N\left(0, {\sigma }^{2}\right)$.

*Elastic net* is a regularized regression method that incorporates the linear combination of ${L}_{1}$ and ${L}_{2}$ penalties of the lasso and ridge methods [12]. The estimates can be obtained from ${\underset{\beta , \alpha }{{\text{argmin}}}\Vert y-\left({\beta }^{T}Z+{\alpha }^{T}X\right)\Vert }^{2}+{\lambda }_{1}\left(\frac{\left(1-{\lambda }_{2}\right)}{2}{\Vert \beta \Vert }_{2}^{2}+{\lambda }_{2}{\Vert \beta \Vert }_{1}\right),$ where tuning parameters ${\lambda }_{1}$ and ${\lambda }_{2}$ can be determined by cross validation (CV). Note that ${\lambda }_{2}=0$ and 1 yield ridge and lasso regressions, respectively. We used the R package ‘glmnet’ [46].

*WQS regression* can be specified as ${Y}_{i}=\psi \left({w}^{T}{\overline{Z} }_{i}\right)+{\alpha }^{T}{X}_{i}+{\epsilon }_{i}$, where $\overline{Z }$ is a pre-specified quantized variable of exposure $Z$. $\psi$ represents the coefficient for the overall linear effect of the mixture, and $w$ is the weight of each exposure. This method assumes that sum of all weights is 1 and each weight is between 0 and 1. Furthermore, this method assumes the same direction in all exposures (i.e., unidirectionality assumption). To conduct the WQS regression, we used ‘gWQS’ R package [17].

*BKMR* includes a completely nonparametric function of exposures as ${Y}_{i}=h\left({Z}_{1i}, \dots , {Z}_{pi}\right)+{\alpha }^{T}{X}_{i}+{\epsilon }_{i}$, where $h\left(\cdot\right)$ characterizes a high-dimensional exposure–response function that may incorporate non-linearity and/or interaction among the mixture components. BKMR provides the posterior inclusion probabilities for each exposure, plotting the exposure–response function, and the cumulative (or overall) effects of the mixture. The R package ‘bkmr’ was used for the analysis [47].

## Appendix B. Algorithm for LOD accommodation using the AFT model (F-AFT)

Using the same notations from Appendix A, the following steps are performed to produce imputation values from the multivariate AFT model:

- *Step 1*. Apply a monotone decreasing transformation ${h}^{-1}\left(\cdot \right)$ to rewrite left-censored $Z$ as right-censored $T$ (e.g., $T={h}^{-1}\left(Z\right)=-{\text{log}}\left(Z\right)$).
- *Step 2*. Fit the AFT model with a normal distribution for each exposure, ${Z}_{j} \left(j=1,\dots ,p\right)$, subject to LOD, where ${T}_{j}={h}^{-1}\left({Z}_{j}\right)={\eta }_{j}^{T}{X}_{j}+{\epsilon }_{j}$. Note that we use the estimate residuals ${\widehat{\epsilon }}_{j}$ to estimate the parameter $\Sigma$ where $\epsilon ={\left({\epsilon }_{1},\dots , {\epsilon }_{p}\right)}^{T} \sim MVN\left(0,\Sigma \right)$
- *Step 3*. Using the estimates from Step 2, obtain the conditional truncated multivariate normal distribution [59–61] for the $i$ th subject, ${\epsilon }_{i}^{\left(c\right)} \sim {f}_{\left(c\right)|\left(o\right), {\epsilon }_{i}^{\left(c\right)}>{\widehat{\epsilon }}_{i}^{(c)}}$ where $\left(c\right)$ and $\left(o\right)$ indicate the vector for the index of the censored variables and observed variables in $Z$, respectively, and ${f}_{\left(c\right), \left(o\right)}$ is the multivariate normal distribution with mean zero and covariance $\widehat{\Sigma }$. For implementation, we used *mtmvnorm* function in ‘tmvtnorm’ R package [62].
- *Step 4*. Impute ${Z}_{i}^{imp}=h\left({T}_{i}^{imp}\right)=h\left({\widehat{\eta }}^{T}{X}_{i}+{\widehat{\epsilon }}_{i}^{imp}\right)$, where ${\widehat{\epsilon }}_{i}^{imp}$ is the conditional expectation of ${f}_{\left(c\right)|\left(o\right), {\epsilon }_{i}^{\left(c\right)}>{\widehat{\epsilon }}_{i}^{(c)}}$ for each subject.

## Appendix C. Algorithm for multiple imputation using bootstrap linear regression

Using the notations from Appendix A, the following procedures are performed to produce imputation values from conventional MI:

- *Step 1*. Draw a bootstrap sample from observed samples.
- *Step 2*. Obtain estimates from linear regression for each exposure $Z$ subject to LOD, $Z={\gamma }^{T}W+{\epsilon }_{MI}$ with ${\epsilon }_{MI} \sim N(0, {\sigma }_{MI}^{2})$. Note that $W$ includes all available variables including the outcome.
- *Step 3*. Draw imputed values ${Z}_{imp} \sim N\left({\widehat{\gamma }}^{T}W, {\widehat{\sigma }}_{MI}^{2}\right)$.

Note that this approach can be easily generalized to multiple exposures using multivariate imputation by chained equations (see Sect. 4.5.2 of Van Buuran [43]). In the truncated MI, we draw the imputed values ${Z}_{imp}$ from a truncated normal distribution, $N\left({\widehat{\gamma }}^{T}W, {\widehat{\sigma }}_{MI}^{2}\right)$ within the range based on LOD (e.g., $[0, LOD]$) in *Step 3*.

## References

[1] Aylward LL (2013). Evaluation of biomonitoring data from the CDC National Exposure Report in a risk assessment context: perspectives across chemicals. Environ Health Perspect.

[2] Exley K (2015). Pilot study testing a European human biomonitoring framework for biomarkers of chemical exposure in children and their mothers: experiences in the UK. Environ Sci Pollut Res.

[3] Frederiksen H (2014). Human urinary excretion of non-persistent environmental chemicals: an overview of Danish data collected between 2006 and 2012. Reproduction.

[4] Hamra GB, Buckley JP (2018). Environmental exposure mixtures: questions and methods to address them. Current epidemiology reports.

[5] Kortenkamp A, Ten,  (2007). years of mixing cocktails: a review of combination effects of endocrine-disrupting chemicals. Environ Health Perspect.

[6] Braun JM (2016). What can epidemiological studies tell us about the impact of chemical mixtures on human health?. Environ Health Perspect.

[7] Kelley AS (2019). Early pregnancy exposure to endocrine disrupting chemical mixtures are associated with inflammatory changes in maternal and neonatal circulation. Sci Rep.

[8] Carrico C (2015). Characterization of weighted quantile sum regression for highly correlated data in a risk analysis setting. J Agric Biol Environ Stat.

[9] Gennings C (2013). A cohort study evaluation of maternal PCB exposure related to time to pregnancy in daughters. Environ Health.

[10] Bobb JF (2018). Statistical software for analyzing the health effects of multiple concurrent exposures via Bayesian kernel machine regression. Environ Health.

[11] Bobb JF (2015). Bayesian kernel machine regression for estimating the health effects of multi-pollutant mixtures. Biostatistics.

[12] Zou H, Hastie T (2005). Regularization and variable selection via the elastic net. J R Stat Soc Ser B Stat Methodol.

[13] Tibshirani R (1996). Regression shrinkage and selection via the lasso. J R Stat Soc Ser B Stat Methodol.

[14] Aung MT (2021). Cross-sectional estimation of endogenous biomarker associations with prenatal phenols, phthalates, metals, and polycyclic aromatic hydrocarbons in single-pollutant and mixtures analysis approaches. Environ Health Perspect.

[15] Lenters V (2016). Prenatal phthalate, perfluoroalkyl acid, and organochlorine exposures and term birth weight in three birth cohorts: multi-pollutant models based on elastic net regression. Environ Health Perspect.

[16] Vuong AM (2020). Prenatal exposure to a mixture of persistent organic pollutants (POPs) and child reading skills at school age. Int J Hyg Environ Health.

[17] Renzetti, S., Curtin, P., Just, A. C., Bello, G. and Gennings, C. (2020) gWQS: Generalized Weighted Quantile Sum Regression. R package version 3.0.0. https://CRAN.R-project.org/package=gWQS. Accessed 8 Oct 2021.

[18] Christensen KLY (2013). Multiple classes of environmental chemicals are associated with liver disease: NHANES 2003–2004. Int J Hyg Environ Health.

[19] Colicino E (2020). Per-and poly-fluoroalkyl substances and bone mineral density: results from the Bayesian weighted quantile sum regression. Environmental Epidemiology.

[20] Czarnota J, Gennings C, Wheeler DC (2015). Assessment of weighted quantile sum regression for modeling chemical mixtures and cancer risk. Cancer Inform..

[21] Domingo-Relloso A (2019). The association of urine metals and metal mixtures with cardiovascular incidence in an adult population from Spain: the Hortega Follow-Up Study. Int J Epidemiol.

[22] Tanner E, Lee A, Colicino E (2020). Environmental mixtures and children's health: identifying appropriate statistical approaches. Curr Opin Pediatr.

[23] Gibson EA (2019). An overview of methods to address distinct research questions on environmental mixtures: an application to persistent organic pollutants and leukocyte telomere length. Environ Health.

[24] Aubert G, Lansdorp PM (2008). Telomeres and aging. Physiol Rev.

[25] Haycock, P.C., et al., Leucocyte telomere length and risk of cardiovascular disease: systematic review and meta-analysis. BMJ. 2014;349:g4227. 10.1136/bmj.g4227 PMC4086028 25006006

[26] Révész D (2014). Telomere length as a marker of cellular aging is associated with prevalence and progression of metabolic syndrome. J Clin Endocrinol Metab.

[27] Willeit P (2014). Leucocyte telomere length and risk of type 2 diabetes mellitus: new prospective cohort study and literature-based meta-analysis. PLoS ONE.

[28] Lan Q (2009). A prospective study of telomere length measured by monochrome multiplex quantitative PCR and risk of non-Hodgkin lymphoma. Clin Cancer Res.

[29] Sanchez-Espiridion B (2014). Telomere length in peripheral blood leukocytes and lung cancer risk: a large case–control study in Caucasians. Can Res.

[30] Seow WJ (2014). Telomere length in white blood cell DNA and lung cancer: a pooled analysis of three prospective cohorts. Can Res.

[31] Xie H (2013). Long telomeres in peripheral blood leukocytes are associated with an increased risk of soft tissue sarcoma. Cancer.

[32] Mitro SD (2016). Cross-sectional associations between exposure to persistent organic pollutants and leukocyte telomere length among US adults in NHANES, 2001–2002. Environ Health Perspect.

[33] Ortega-Villa AM (2021). New insights into modeling exposure measurements below the limit of detection. Environmental Epidemiology.

[34] Little RJ (1992). Regression with missing X's: a review. J Am Stat Assoc.

[35] Little, R.J. and D.B. Rubin, Statistical analysis with missing data. Vol. 793. Hoboken: Wiley; 2019.

[36] D'Angelo G, Weissfeld L, Investigators G (2008). An index approach for the Cox model with left censored covariates. Stat Med.

[37] Nie L (2010). Linear regression with an independent variable subject to a detection limit. Epidemiology.

[38] Richardson DB, Ciampi A (2003). Effects of exposure measurement error when an exposure variable is constrained by a lower limit. Am J Epidemiol.

[39] Schisterman EF (2006). The limitations due to exposure detection limits for regression models. Am J Epidemiol.

[40] Helsel D: Nondetects and data analysis: statistics for censored environmental data. Hoboken: Wiley-Interscience; 2005.

[41] Chen LW (2022). Semiparametric analysis of a generalized linear model with multiple covariates subject to detection limits. Stat Med.

[42] Kong S, Nan B (2016). Semiparametric approach to regression with a covariate subject to a detection limit. Biometrika.

[43] Van Buuren, S. van. Flexible Imputation of Missing Data, Second Edition. (Chapman and Hall/CRC, New York, 2018). 10.1201/9780429492259.

[44] Little RJA, Rubin DB: Statistical Analysis with Missing Data. New York: Wiley; 1987.

[45] Van Buuren S, Groothuis-Oudshoorn K (2011). mice: Multivariate imputation by chained equations in R. J Stat Softw.

[46] Friedman J, Hastie T, Tibshirani R, Narasimhan B, Tay K, Simon N, Qian J. Package ‘glmnet’. J Stat Softw. 2022;33(1):2010a.

[47] Bobb JF. bkmr: Bayesian Kernel Machine Regression. R package version 0.2.0; Published online 2017.

[48] Blei, D.M. (2015) Regularized regression. Columbia University, New York, 1–11. http://www.cs.columbia.edu/~blei/fogm/2015F/notes/regularized-regression.pdf.

[49] Zipf G, Chiappa M, Porter KS, Ostchega Y, Lewis BG, Dostal J. Health and nutrition examination survey planand operations, 1999-2010. Vital Health Stat. 2013;1(56). 25078429

[50] U.S. CDC (U.S. Centers for Disease Control and Prevention). 2002. Laboratory Procedure Manual: PCBs and Persistent Pesticides in Serum. 2001–2002. Atlanta, GA: U.S. CDC. https://www.cdc.gov/nchs/data/nhanes/nhanes_01_02/l28poc_b_met_pcb_pesticides.pdf. Accessed 7 Sept 2021.

[51] Centers for Disease Control and Prevention. 2006. Laboratory Procedure Manual: PCBs and PersistentPesticides. Available: http://www.cdc.gov/nchs/data/nhanes/nhanes_03_04/l28_c_met_%20PCBs_and_Persistent_Pesticides.pdf.Accessed 23 Apr 2009.

[52] Chiou SH, Betensky RA, Balasubramanian R (2019). The missing indicator approach for censored covariates subject to limit of detection in logistic regression models. Ann Epidemiol.

[53] Zhang W (2020). Nonparametric estimation of distributions and diagnostic accuracy based on group-tested results with differential misclassification. Biometrics.

[54] Bloom MS (2021). Association between gestational phthalate exposure and newborn head circumference; impacts by race and sex. Environ Res.

[55] Kim SS (2018). Urinary trace metals individually and in mixtures in association with preterm birth. Environ Int.

[56] Trowbridge J (2023). Extending nontargeted discovery of environmental chemical exposures during pregnancy and their association with pregnancy complications—a cross-sectional study. Environ Health Perspect.

[57] Keil AP (2020). A quantile-based g-computation approach to addressing the effects of exposure mixtures. Environ Health Perspect.

[58] Robins J (1986). A new approach to causal inference in mortality studies with a sustained exposure period—application to control of the healthy worker survivor effect. Mathematical modelling.

[59] Lee L-F (1979). On the first and second moments of the truncated multi-normal distribution and a simple estimator. Econ Lett.

[60] Leppard P, Tallis G (1989). Algorithm AS 249: Evaluation of the mean and covariance of the truncated multinormal distribution. J R Stat Soc  Society Series C (Applied Statistics).

[61] Tallis GM (1961). The moment generating function of the truncated multi-normal distribution. J R Stat Soc Ser B Stat Methodol.

[62] Wilhelm S, Manjunath BG, 2015. Package ’tmvtnorm’: Truncated Multivariate Normal and Student t Distribution, TMVTNORM: Truncated Multivariate Normal and Student t Distribution. Available from CRAN.Rproject.org/package=tmvtnorm. last accessed May 2024.

