# Supplementary material for: Accommodating detection limits of multiple exposures in environmental mixture analyses: an overview of statistical approaches
Source: Environ Health. 2024 May 16;23:48. doi: 10.1186/s12940-024-01088-w (PMC11097582; doi:10.1186/s12940-024-01088-w)
Supplement: Supplementary file 1 — Supplementary Material 1. [file 12940_2024_1088_MOESM1_ESM.docx]

**Table S1. Elastic net: bias (SE) and** $\boldsymbol{R}^{\boldsymbol{2}}$ **of each LOD accommodation approach compared to using full dataset.**

| **Scenario 1** | Complete case | LOD/$\sqrt{2}$ | MI | Truncated MI | F-AFT |
| --- | --- | --- | --- | --- | --- |
|  | Moderate correlation ($\sigma=1/2$) | | | | |
| $\beta_{1}$ | -0.12 (0.26) | 0.03 (0.14) | 0.05 (0.14) | 0.00 (0.14) | 0.02 (0.14) |
| $\beta_{2}$ | -0.13 (0.28) | -0.03 (0.14) | 0.02 (0.20) | 0.01 (0.14) | 0.01 (0.15) |
| $\beta_{3}$ | 0.02 (0.21) | 0.01 (0.11) | 0.02 (0.20) | 0.00 (0.12) | 0.00 (0.12) |
| $\beta_{4}$ | -0.10 (0.32) | 0.03 (0.19) | 0.04 (0.21) | 0.00 (0.20) | 0.04 (0.19) |
| $\beta_{5}$ | -0.07 (0.32) | -0.05 (0.18) | 0.01 (0.27) | 0.01 (0.21) | -0.02 (0.20) |
| $\beta_{6}$ | 0.04 (0.23) | 0.02 (0.15) | 0.03 (0.16) | 0.00 (0.16) | 0.02 (0.15) |
| $\beta_{7}$ | -0.03 (0.24) | -0.01 (0.13) | 0.01 (0.20) | 0.01 (0.14) | 0.00 (0.14) |
| $\beta_{8}$ | 0.00 (0.19) | 0.01 (0.12) | 0.02 (0.13) | 0.00 (0.13) | 0.01 (0.12) |
| $\beta_{9}$ | -0.01 (0.19) | -0.02 (0.11) | -0.01 (0.17) | 0.00 (0.12) | 0.00 (0.11) |
| $\beta_{10}$ | 0.00 (0.17) | 0.01 (0.11) | 0.01 (0.12) | 0.00 (0.11) | 0.00 (0.11) |
| $\alpha_{0}$ | 0.29 (0.52) | -0.07 (0.15) | -0.46 (0.27) | -0.01 (0.15) | -0.06 (0.16) |
| $\alpha_{1}$ | 0.07 (0.21) | 0.06 (0.10) | 0.10 (0.11) | -0.01 (0.11) | 0.00 (0.11) |
| $\alpha_{2}$ | 0.02 (0.28) | 0.01 (0.15) | 0.00 (0.15) | 0.00 (0.15) | 0.00 (0.15) |
| $R^{2}$ | 0.74 | 0.84 | 0.80 | 0.83 | 0.84 |
|  | High correlation ($\sigma=1/8$) | | | | |
| $\beta_{1}$ | -0.17 (0.64) | -0.03 (0.53) | 0.06 (0.52) | 0.04 (0.52) | 0.02 (0.53) |
| $\beta_{2}$ | -0.12 (0.41) | -0.16 (0.23) | 0.12 (0.50) | 0.08 (0.46) | -0.06 (0.41) |
| $\beta_{3}$ | -0.03 (0.42) | -0.04 (0.22) | -0.03 (0.53) | -0.01 (0.40) | -0.01 (0.34) |
| $\beta_{4}$ | -0.10 (0.64) | 0.00 (0.47) | 0.04 (0.57) | 0.02 (0.53) | 0.02 (0.50) |
| $\beta_{5}$ | -0.06 (0.66) | -0.16 (0.26) | 0.06 (0.73) | 0.03 (0.59) | -0.03 (0.49) |
| $\beta_{6}$ | -0.03 (0.55) | 0.02 (0.37) | 0.00 (0.49) | 0.00 (0.46) | 0.02 (0.41) |
| $\beta_{7}$ | -0.01 (0.42) | -0.04 (0.20) | 0.03 (0.50) | 0.03 (0.41) | -0.01 (0.33) |
| $\beta_{8}$ | 0.00 (0.36) | 0.01 (0.28) | 0.01 (0.34) | 0.00 (0.34) | 0.00 (0.30) |
| $\beta_{9}$ | -0.02 (0.30) | -0.04 (0.18) | 0.00 (0.39) | 0.01 (0.33) | -0.01 (0.22) |
| $\beta_{10}$ | -0.01 (0.37) | 0.01 (0.26) | 0.00 (0.35) | -0.01 (0.32) | 0.01 (0.29) |
| $\alpha_{0}$ | 0.23 (0.58) | 0.16 (0.29) | -0.15 (0.44) | -0.05 (0.35) | 0.02 (0.36) |
| $\alpha_{1}$ | 0.12 (0.37) | 0.12 (0.24) | -0.06 (0.34) | -0.06 (0.34) | 0.03 (0.33) |
| $\alpha_{2}$ | 0.03 (0.32) | 0.03 (0.25) | -0.03 (0.26) | -0.02 (0.26) | 0.00 (0.26) |
| $R^{2}$ | 0.60 | 0.79 | 0.82 | 0.85 | 0.84 |
| **Scenario 2A** | Complete case | LOD/$\sqrt{2}$ | MI | Truncated MI | F-AFT |
|  | Moderate correlation ($\sigma=1/2$) | | | | |
| $\beta_{1}$ | 0.00 (0.26) | 0.14 (0.14) | 0.15 (0.15) | 0.15 (0.15) | 0.14 (0.14) |
| $\beta_{3}$ | 0.09 (0.22) | 0.10 (0.13) | 0.12 (0.20) | 0.13 (0.20) | 0.11 (0.14) |
| $\beta_{4}$ | -0.12 (0.30) | 0.02 (0.19) | 0.03 (0.20) | -0.01 (0.20) | 0.03 (0.19) |
| $\beta_{5}$ | -0.07 (0.31) | -0.06 (0.18) | 0.01 (0.27) | 0.00 (0.21) | -0.03 (0.20) |
| $\beta_{6}$ | 0.04 (0.22) | 0.03 (0.15) | 0.03 (0.16) | 0.00 (0.15) | 0.03 (0.15) |
| $\beta_{7}$ | -0.03 (0.23) | -0.01 (0.13) | 0.01 (0.20) | -0.01 (0.13) | 0.00 (0.14) |
| $\beta_{8}$ | -0.01 (0.18) | 0.00 (0.12) | 0.01 (0.13) | 0.00 (0.12) | 0.00 (0.12) |
| $\beta_{9}$ | -0.01 (0.17) | 0.00 (0.11) | 0.00 (0.17) | 0.00 (0.11) | 0.00 (0.11) |
| $\beta_{10}$ | 0.00 (0.16) | 0.01 (0.11) | 0.01 (0.11) | 0.00 (0.11) | 0.00 (0.10) |
| $\alpha_{0}$ | 0.41 (0.46) | 0.19 (0.15) | -0.01 (0.24) | 0.14 (0.18) | 0.16 (0.15) |
| $\alpha_{1}$ | 0.34 (0.17) | 0.31 (0.10) | 0.31 (0.10) | 0.31 (0.10) | 0.30 (0.10) |
| $\alpha_{2}$ | 0.04 (0.27) | 0.02 (0.15) | 0.01 (0.16) | 0.01 (0.15) | 0.01 (0.15) |
| $R^{2}$ | 0.52 | 0.62 | 0.61 | 0.61 | 0.62 |
|  | High correlation ($\sigma=1/8$) | | | | |
| $\beta_{1}$ | -0.17 (0.65) | -0.01 (0.54) | 0.09 (0.55) | 0.07 (0.54) | 0.03 (0.54) |
| $\beta_{3}$ | 0.00 (0.40) | -0.02 (0.21) | 0.05 (0.50) | 0.04 (0.48) | 0.01 (0.32) |
| $\beta_{4}$ | -0.11 (0.61) | 0.00 (0.46) | 0.02 (0.55) | 0.00 (0.51) | 0.01 (0.49) |
| $\beta_{5}$ | -0.08 (0.64) | -0.16 (0.26) | 0.06 (0.70) | 0.02 (0.56) | -0.04 (0.47) |
| $\beta_{6}$ | -0.02 (0.54) | 0.02 (0.36) | 0.00 (0.47) | -0.01 (0.44) | 0.01 (0.38) |
| $\beta_{7}$ | -0.02 (0.41) | -0.03 (0.20) | 0.03 (0.48) | 0.02 (0.34) | -0.02 (0.30) |
| $\beta_{8}$ | 0.00 (0.34) | 0.00 (0.28) | 0.01 (0.33) | 0.00 (0.31) | 0.00 (0.29) |
| $\beta_{9}$ | -0.01 (0.29) | -0.01 (0.16) | 0.00 (0.38) | 0.00 (0.26) | -0.01 (0.21) |
| $\beta_{10}$ | -0.01 (0.36) | 0.00 (0.26) | 0.00 (0.33) | -0.01 (0.29) | 0.01 (0.28) |
| $\alpha_{0}$ | 0.28 (0.55) | 0.20 (0.26) | 0.00 (0.38) | 0.07 (0.34) | 0.10 (0.31) |
| $\alpha_{1}$ | 0.20 (0.29) | 0.17 (0.21) | 0.12 (0.25) | 0.12 (0.23) | 0.15 (0.23) |
| $\alpha_{2}$ | 0.04 (0.31) | 0.04 (0.24) | -0.01 (0.25) | 0.00 (0.25) | 0.01 (0.25) |
| $R^{2}$ | 0.56 | 0.77 | 0.77 | 0.78 | 0.80 |
| **Scenario 2B** | Complete case | LOD/$\sqrt{2}$ | MI | Truncated MI | F-AFT |
|  | Moderate correlation ($\sigma=1/2$) | | | | |
| $\beta_{1}$ | -0.04 (0.24) | 0.09 (0.14) | 0.10 (0.14) | 0.09 (0.14) | 0.09 (0.14) |
| $\beta_{2}$ | -0.26 (0.26) | -0.18 (0.17) | -0.17 (0.17) | -0.17 (0.17) | -0.18 (0.17) |
| $\beta_{3}$ | 0.06 (0.21) | 0.06 (0.12) | 0.06 (0.20) | 0.07 (0.19) | 0.06 (0.13) |
| $\beta_{4}$ | -0.10 (0.30) | 0.03 (0.19) | 0.04 (0.20) | -0.01 (0.20) | 0.03 (0.19) |
| $\beta_{5}$ | -0.06 (0.31) | -0.05 (0.18) | 0.01 (0.27) | 0.01 (0.21) | -0.02 (0.20) |
| $\beta_{6}$ | 0.04 (0.22) | 0.03 (0.15) | 0.03 (0.16) | 0.00 (0.16) | 0.03 (0.15) |
| $\beta_{7}$ | -0.03 (0.23) | -0.01 (0.13) | 0.02 (0.20) | 0.00 (0.13) | 0.00 (0.14) |
| $\beta_{8}$ | 0.00 (0.19) | 0.01 (0.12) | 0.01 (0.13) | 0.00 (0.12) | 0.01 (0.12) |
| $\beta_{9}$ | -0.01 (0.18) | -0.01 (0.11) | 0.00 (0.17) | 0.00 (0.11) | 0.00 (0.11) |
| $\beta_{10}$ | 0.00 (0.16) | 0.01 (0.11) | 0.01 (0.11) | 0.00 (0.11) | 0.01 (0.11) |
| $\alpha_{0}$ | 0.42 (0.44) | 0.18 (0.15) | 0.00 (0.23) | 0.16 (0.18) | 0.15 (0.15) |
| $\alpha_{1}$ | 0.21 (0.18) | 0.20 (0.10) | 0.20 (0.10) | 0.19 (0.10) | 0.19 (0.10) |
| $\alpha_{2}$ | 0.03 (0.26) | 0.01 (0.15) | 0.01 (0.15) | 0.00 (0.15) | 0.00 (0.15) |
| $R^{2}$ | 0.64 | 0.73 | 0.73 | 0.73 | 0.74 |
|  | High correlation ($\sigma=1/8$) | | | | |
| $\beta_{1}$ | -0.19 (0.63) | -0.05 (0.54) | 0.05 (0.54) | 0.03 (0.54) | -0.01 (0.54) |
| $\beta_{2}$ | -0.27 (0.14) | -0.26 (0.12) | -0.25 (0.14) | -0.26 (0.13) | -0.26 (0.13) |
| $\beta_{3}$ | -0.01 (0.39) | -0.02 (0.20) | 0.03 (0.48) | 0.02 (0.46) | 0.01 (0.31) |
| $\beta_{4}$ | -0.12 (0.58) | -0.02 (0.45) | 0.00 (0.53) | -0.02 (0.49) | -0.01 (0.47) |
| $\beta_{5}$ | -0.08 (0.60) | -0.16 (0.25) | 0.06 (0.67) | 0.01 (0.54) | -0.05 (0.46) |
| $\beta_{6}$ | -0.02 (0.50) | 0.02 (0.35) | -0.01 (0.44) | -0.01 (0.41) | 0.01 (0.36) |
| $\beta_{7}$ | -0.02 (0.39) | -0.04 (0.20) | 0.02 (0.46) | 0.01 (0.32) | -0.02 (0.28) |
| $\beta_{8}$ | 0.00 (0.32) | 0.00 (0.26) | 0.01 (0.32) | 0.00 (0.30) | 0.01 (0.27) |
| $\beta_{9}$ | -0.02 (0.27) | -0.01 (0.15) | 0.00 (0.36) | 0.00 (0.24) | -0.01 (0.19) |
| $\beta_{10}$ | -0.02 (0.34) | 0.00 (0.24) | 0.00 (0.31) | -0.01 (0.27) | 0.00 (0.26) |
| $\alpha_{0}$ | 0.30 (0.54) | 0.22 (0.26) | 0.03 (0.38) | 0.10 (0.33) | 0.13 (0.31) |
| $\alpha_{1}$ | 0.20 (0.29) | 0.17 (0.21) | 0.12 (0.25) | 0.13 (0.23) | 0.15 (0.23) |
| $\alpha_{2}$ | 0.04 (0.30) | 0.05 (0.24) | 0.00 (0.25) | 0.01 (0.25) | 0.03 (0.25) |
| $R^{2}$ | 0.54 | 0.76 | 0.76 | 0.78 | 0.80 |
| **Scenario 3** | Complete case | LOD/$\sqrt{2}$ | MI | Truncated MI | F-AFT |
|  | Moderate correlation ($\sigma=1/2$) | | | | |
| $\beta_{1}$ | -0.10 (0.26) | 0.00 (0.14) | 0.07 (0.15) | -0.03 (0.14) | 0.00 (0.14) |
| $\beta_{2}$ | -0.28 (0.30) | 0.13 (0.15) | -0.03 (0.22) | 0.18 (0.15) | 0.15 (0.15) |
| $\beta_{3}$ | 0.01 (0.21) | -0.01 (0.11) | 0.03 (0.20) | -0.02 (0.12) | -0.02 (0.12) |
| $\beta_{4}$ | -0.10 (0.31) | 0.04 (0.20) | 0.04 (0.21) | 0.00 (0.21) | 0.04 (0.20) |
| $\beta_{5}$ | -0.06 (0.31) | -0.05 (0.20) | 0.02 (0.29) | 0.01 (0.22) | -0.02 (0.22) |
| $\beta_{6}$ | 0.03 (0.23) | 0.02 (0.16) | 0.02 (0.17) | -0.01 (0.16) | 0.02 (0.16) |
| $\beta_{7}$ | -0.03 (0.23) | -0.01 (0.13) | 0.02 (0.21) | 0.01 (0.14) | 0.00 (0.14) |
| $\beta_{8}$ | -0.01 (0.18) | 0.01 (0.12) | 0.02 (0.14) | 0.00 (0.12) | 0.00 (0.12) |
| $\beta_{9}$ | 0.00 (0.18) | -0.01 (0.11) | -0.01 (0.17) | 0.00 (0.12) | 0.00 (0.11) |
| $\beta_{10}$ | 0.00 (0.18) | 0.01 (0.11) | 0.01 (0.12) | 0.00 (0.12) | 0.00 (0.11) |
| $\alpha_{0}$ | 0.56 (0.56) | -0.14 (0.16) | -0.48 (0.28) | -0.05 (0.16) | -0.11 (0.16) |
| $\alpha_{1}$ | 0.06 (0.21) | 0.01 (0.11) | 0.13 (0.12) | -0.08 (0.12) | -0.06 (0.12) |
| $\alpha_{2}$ | 0.02 (0.27) | 0.00 (0.15) | 0.00 (0.16) | 0.00 (0.15) | 0.00 (0.15) |
| $R^{2}$ | 0.71 | 0.85 | 0.78 | 0.83 | 0.84 |
|  | High correlation ($\sigma=1/8$) | | | | |
| $\beta_{1}$ | -0.17 (0.35) | 0.00 (0.14) | 0.07 (0.15) | -0.04 (0.14) | 0.00 (0.14) |
| $\beta_{2}$ | -0.34 (0.35) | 0.16 (0.15) | 0.00 (0.25) | 0.22 (0.16) | 0.19 (0.16) |
| $\beta_{3}$ | 0.02 (0.26) | -0.01 (0.11) | 0.04 (0.24) | -0.03 (0.12) | -0.03 (0.12) |
| $\beta_{4}$ | -0.15 (0.35) | 0.06 (0.20) | 0.04 (0.21) | 0.00 (0.21) | 0.06 (0.20) |
| $\beta_{5}$ | -0.07 (0.38) | -0.06 (0.20) | 0.04 (0.35) | 0.02 (0.24) | -0.04 (0.23) |
| $\beta_{6}$ | 0.03 (0.27) | 0.03 (0.16) | 0.03 (0.18) | -0.01 (0.17) | 0.03 (0.16) |
| $\beta_{7}$ | -0.04 (0.28) | -0.01 (0.14) | 0.03 (0.26) | 0.01 (0.15) | -0.01 (0.15) |
| $\beta_{8}$ | -0.01 (0.21) | 0.01 (0.12) | 0.02 (0.14) | 0.00 (0.13) | 0.01 (0.12) |
| $\beta_{9}$ | 0.00 (0.21) | -0.01 (0.11) | -0.01 (0.20) | 0.00 (0.13) | 0.00 (0.12) |
| $\beta_{10}$ | -0.01 (0.21) | 0.01 (0.11) | 0.01 (0.13) | 0.00 (0.12) | 0.00 (0.11) |
| $\alpha_{0}$ | 0.73 (0.75) | -0.21 (0.17) | -0.62 (0.35) | -0.06 (0.16) | -0.14 (0.17) |
| $\alpha_{1}$ | 0.10 (0.26) | 0.01 (0.11) | 0.13 (0.13) | -0.10 (0.12) | -0.07 (0.12) |
| $\alpha_{2}$ | 0.03 (0.35) | 0.01 (0.15) | 0.00 (0.16) | -0.01 (0.15) | -0.01 (0.15) |
| $R^{2}$ | 0.65 | 0.86 | 0.77 | 0.83 | 0.84 |
| **Scenario 4** | Complete case | LOD/$\sqrt{2}$ | MI | Truncated MI | F-AFT |
|  | Moderate correlation ($\sigma=1/2$) | | | | |
| $\beta_{1}$ | -0.12 (0.26) | 0.00 (0.14) | 0.04 (0.14) | -0.02 (0.14) | 0.00 (0.14) |
| $\beta_{2}$ | -0.10 (0.30) | 0.10 (0.15) | 0.06 (0.22) | 0.12 (0.15) | 0.12 (0.16) |
| $\beta_{3}$ | 0.02 (0.22) | 0.00 (0.11) | 0.03 (0.18) | -0.01 (0.11) | -0.01 (0.12) |
| $\beta_{4}$ | -0.12 (0.32) | 0.03 (0.19) | 0.04 (0.20) | 0.00 (0.20) | 0.04 (0.19) |
| $\beta_{5}$ | -0.05 (0.34) | -0.05 (0.18) | 0.01 (0.27) | 0.01 (0.21) | -0.02 (0.20) |
| $\beta_{6}$ | 0.02 (0.25) | 0.02 (0.15) | 0.03 (0.16) | 0.00 (0.15) | 0.02 (0.15) |
| $\beta_{7}$ | -0.03 (0.23) | -0.01 (0.13) | 0.02 (0.20) | 0.01 (0.14) | 0.00 (0.14) |
| $\beta_{8}$ | -0.01 (0.18) | 0.01 (0.12) | 0.01 (0.12) | 0.00 (0.12) | 0.01 (0.12) |
| $\beta_{9}$ | 0.00 (0.19) | 0.00 (0.11) | 0.00 (0.17) | 0.00 (0.12) | 0.00 (0.11) |
| $\beta_{10}$ | -0.01 (0.18) | 0.01 (0.11) | 0.01 (0.12) | 0.00 (0.12) | 0.00 (0.11) |
| $\alpha_{0}$ | 0.28 (0.55) | -0.12 (0.15) | -0.48 (0.26) | -0.04 (0.14) | -0.09 (0.15) |
| $\alpha_{1}$ | 0.06 (0.20) | 0.00 (0.10) | 0.08 (0.12) | -0.05 (0.11) | -0.05 (0.11) |
| $\alpha_{2}$ | 0.02 (0.28) | 0.00 (0.14) | 0.01 (0.15) | 0.00 (0.15) | -0.01 (0.15) |
| $R^{2}$ | 0.74 | 0.86 | 0.81 | 0.84 | 0.85 |
|  | High correlation ($\sigma=1/8$) | | | | |
| $\beta_{1}$ | -0.19 (0.32) | 0.00 (0.13) | 0.05 (0.14) | -0.02 (0.13) | 0.00 (0.13) |
| $\beta_{2}$ | -0.18 (0.35) | 0.14 (0.15) | 0.06 (0.23) | 0.15 (0.15) | 0.15 (0.16) |
| $\beta_{3}$ | 0.03 (0.28) | 0.00 (0.11) | 0.03 (0.22) | -0.02 (0.12) | -0.02 (0.12) |
| $\beta_{4}$ | -0.17 (0.36) | 0.05 (0.18) | 0.05 (0.20) | 0.00 (0.20) | 0.06 (0.18) |
| $\beta_{5}$ | -0.07 (0.40) | -0.06 (0.18) | 0.03 (0.32) | 0.01 (0.22) | -0.04 (0.20) |
| $\beta_{6}$ | 0.03 (0.29) | 0.03 (0.15) | 0.03 (0.17) | 0.00 (0.16) | 0.03 (0.15) |
| $\beta_{7}$ | -0.03 (0.28) | -0.01 (0.14) | 0.03 (0.23) | 0.01 (0.15) | 0.00 (0.15) |
| $\beta_{8}$ | -0.01 (0.21) | 0.01 (0.12) | 0.02 (0.12) | 0.00 (0.12) | 0.01 (0.12) |
| $\beta_{9}$ | -0.01 (0.22) | -0.01 (0.11) | 0.00 (0.20) | -0.01 (0.13) | -0.01 (0.11) |
| $\beta_{10}$ | 0.00 (0.22) | 0.01 (0.11) | 0.01 (0.12) | 0.00 (0.12) | 0.01 (0.11) |
| $\alpha_{0}$ | 0.46 (0.77) | -0.18 (0.16) | -0.59 (0.32) | -0.05 (0.14) | -0.12 (0.16) |
| $\alpha_{1}$ | 0.10 (0.26) | 0.01 (0.10) | 0.09 (0.12) | -0.06 (0.11) | -0.06 (0.12) |
| $\alpha_{2}$ | 0.04 (0.36) | 0.01 (0.15) | 0.01 (0.15) | -0.01 (0.15) | -0.01 (0.15) |
| $R^{2}$ | 0.67 | 0.86 | 0.79 | 0.84 | 0.85 |

**Abbreviations**: Imputation by LOD/$\sqrt{2}$ (LOD/$\sqrt{2}$); conventional multiple imputation (MI); truncated multiple imputation (Truncated MI); imputation by estimates using the AFT model (F-AFT).

*Note*: $\beta_{j}$ is the coefficient for each exposure $Z_{j} (j=1,\ldots,10)$; $\alpha_{k}$ is the coefficient for intercept, $X_{1}$ and $X_{2}$ $\left( k=0,1,2 \right)$; and $R^{2}$ was calculated by regression $\hat{h}$ from each LOD accommodation on $\hat{h}$ with the full dataset.

**Table S2. WQS: bias (SE) and** $\boldsymbol{R}^{\boldsymbol{2}}$ **of each LOD accommodation approach compared to using full dataset.**

| **Scenario 1** | Complete case | LOD/$\sqrt{2}$ | MI | Truncated MI | F-AFT |
| --- | --- | --- | --- | --- | --- |
|  | Moderate correlation ($\sigma=1/2$) | | | | |
| $\psi$ | -0.34 (0.33) | 0.15 (0.22) | -0.06 (0.18) | 0.00 (0.18) | -0.01 (0.18) |
| $w_{1}$ | -0.03 (0.10) | -0.02 (0.06) | 0.03 (0.06) | 0.00 (0.06) | 0.00 (0.06) |
| $w_{2}$ | -0.04 (0.10) | 0.00 (0.08) | -0.05 (0.06) | 0.00 (0.07) | 0.00 (0.07) |
| $w_{3}$ | 0.02 (0.06) | 0.01 (0.05) | 0.00 (0.04) | 0.00 (0.04) | 0.00 (0.04) |
| $w_{4}$ | -0.02 (0.08) | 0.00 (0.06) | 0.01 (0.07) | 0.00 (0.06) | 0.00 (0.07) |
| $w_{5}$ | -0.02 (0.07) | 0.00 (0.07) | -0.01 (0.06) | 0.00 (0.06) | 0.00 (0.06) |
| $w_{6}$ | 0.01 (0.06) | 0.00 (0.05) | 0.01 (0.05) | 0.00 (0.05) | 0.00 (0.05) |
| $w_{7}$ | 0.01 (0.06) | 0.01 (0.04) | 0.00 (0.04) | 0.00 (0.04) | 0.00 (0.04) |
| $w_{8}$ | 0.02 (0.06) | 0.00 (0.04) | 0.00 (0.04) | 0.00 (0.04) | 0.00 (0.04) |
| $w_{9}$ | 0.02 (0.06) | 0.00 (0.04) | 0.00 (0.04) | 0.00 (0.03) | 0.00 (0.04) |
| $w_{10}$ | 0.02 (0.05) | 0.00 (0.03) | 0.00 (0.03) | 0.00 (0.03) | 0.00 (0.03) |
| $\alpha_{0}$ | 1.43 (0.51) | 0.31 (0.22) | -0.01 (0.24) | 0.00 (0.23) | 0.04 (0.23) |
| $\alpha_{1}$ | 0.00 (0.25) | 0.06 (0.12) | 0.09 (0.12) | 0.00 (0.13) | -0.01 (0.14) |
| $\alpha_{2}$ | -0.04 (0.35) | 0.01 (0.19) | 0.01 (0.19) | 0.00 (0.19) | -0.02 (0.19) |
| $R^{2}$ | 0.73 | 0.86 | 0.79 | 0.84 | 0.85 |
|  | High correlation ($\sigma=1/8$) | | | | |
| $\psi$ | -0.03 (0.33) | 0.01 (0.26) | 0.01 (0.24) | 0.01 (0.24) | -0.01 (0.24) |
| $w_{1}$ | -0.02 (0.13) | 0.00 (0.13) | 0.00 (0.13) | 0.00 (0.13) | 0.00 (0.13) |
| $w_{2}$ | 0.01 (0.13) | -0.02 (0.11) | 0.00 (0.11) | 0.00 (0.11) | -0.01 (0.11) |
| $w_{3}$ | 0.01 (0.10) | 0.00 (0.10) | 0.00 (0.09) | 0.00 (0.10) | 0.00 (0.10) |
| $w_{4}$ | -0.02 (0.10) | 0.00 (0.10) | 0.00 (0.10) | 0.00 (0.10) | 0.00 (0.11) |
| $w_{5}$ | -0.02 (0.10) | 0.01 (0.11) | 0.00 (0.10) | 0.00 (0.10) | 0.00 (0.10) |
| $w_{6}$ | -0.01 (0.09) | 0.00 (0.09) | 0.00 (0.09) | 0.00 (0.09) | 0.00 (0.09) |
| $w_{7}$ | 0.01 (0.09) | 0.00 (0.09) | 0.01 (0.08) | 0.00 (0.08) | 0.00 (0.09) |
| $w_{8}$ | 0.01 (0.10) | 0.00 (0.09) | 0.00 (0.09) | 0.00 (0.09) | 0.00 (0.10) |
| $w_{9}$ | 0.02 (0.09) | 0.00 (0.07) | 0.00 (0.07) | 0.00 (0.07) | 0.00 (0.07) |
| $w_{10}$ | 0.01 (0.09) | 0.00 (0.08) | 0.00 (0.08) | 0.00 (0.07) | 0.00 (0.08) |
| $\alpha_{0}$ | 0.48 (0.46) | 0.06 (0.16) | -0.01 (0.18) | 0.00 (0.18) | 0.01 (0.18) |
| $\alpha_{1}$ | -0.09 (0.37) | 0.03 (0.18) | -0.01 (0.19) | 0.00 (0.19) | 0.00 (0.20) |
| $\alpha_{2}$ | -0.06 (0.39) | 0.01 (0.26) | -0.01 (0.26) | 0.00 (0.26) | 0.00 (0.26) |
| $R^{2}$ | 0.87 | 0.92 | 0.93 | 0.93 | 0.93 |
| **Scenario 2A** | Complete case | LOD/$\sqrt{2}$ | MI | Truncated MI | F-AFT |
|  | Moderate correlation ($\sigma=1/2$) | | | | |
| $\psi$ | -0.49 (0.29) | -0.17 (0.19) | -0.28 (0.17) | -0.28 (0.17) | -0.28 (0.17) |
| $w_{1}$ | 0.08 (0.12) | 0.08 (0.07) | 0.11 (0.07) | 0.12 (0.07) | 0.11 (0.07) |
| $w_{3}$ | 0.05 (0.08) | 0.05 (0.07) | 0.02 (0.05) | 0.03 (0.05) | 0.04 (0.06) |
| $w_{4}$ | 0.00 (0.09) | 0.03 (0.08) | 0.04 (0.08) | 0.03 (0.08) | 0.03 (0.08) |
| $w_{5}$ | 0.00 (0.08) | 0.03 (0.09) | 0.01 (0.07) | 0.02 (0.08) | 0.02 (0.08) |
| $w_{6}$ | 0.02 (0.08) | 0.01 (0.06) | 0.02 (0.06) | 0.01 (0.06) | 0.02 (0.06) |
| $w_{7}$ | 0.02 (0.06) | 0.02 (0.06) | 0.01 (0.04) | 0.01 (0.05) | 0.01 (0.05) |
| $w_{8}$ | 0.03 (0.06) | 0.01 (0.04) | 0.01 (0.05) | 0.01 (0.05) | 0.01 (0.05) |
| $w_{9}$ | 0.03 (0.06) | 0.02 (0.05) | 0.01 (0.04) | 0.01 (0.04) | 0.01 (0.04) |
| $w_{10}$ | 0.03 (0.06) | 0.01 (0.04) | 0.01 (0.04) | 0.01 (0.04) | 0.01 (0.04) |
| $\alpha_{0}$ | 1.12 (0.46) | 0.35 (0.22) | 0.16 (0.23) | 0.18 (0.23) | 0.21 (0.22) |
| $\alpha_{1}$ | 0.23 (0.22) | 0.24 (0.11) | 0.24 (0.11) | 0.23 (0.11) | 0.22 (0.12) |
| $\alpha_{2}$ | -0.02 (0.34) | 0.02 (0.20) | 0.01 (0.20) | 0.01 (0.20) | -0.01 (0.20) |
| $R^{2}$ | 0.54 | 0.65 | 0.63 | 0.63 | 0.65 |
|  | High correlation ($\sigma=1/8$) | | | | |
| $\psi$ | -0.05 (0.30) | -0.01 (0.25) | -0.01 (0.23) | -0.01 (0.23) | -0.02 (0.23) |
| $w_{1}$ | 0.02 (0.14) | 0.03 (0.14) | 0.03 (0.14) | 0.04 (0.14) | 0.04 (0.14) |
| $w_{3}$ | 0.03 (0.11) | 0.02 (0.11) | 0.02 (0.10) | 0.02 (0.11) | 0.02 (0.12) |
| $w_{4}$ | -0.01 (0.10) | 0.01 (0.11) | 0.01 (0.11) | 0.01 (0.11) | 0.01 (0.12) |
| $w_{5}$ | -0.01 (0.10) | 0.02 (0.12) | 0.01 (0.11) | 0.01 (0.11) | 0.01 (0.11) |
| $w_{6}$ | 0.00 (0.10) | 0.01 (0.10) | 0.01 (0.10) | 0.01 (0.10) | 0.01 (0.10) |
| $w_{7}$ | 0.02 (0.10) | 0.01 (0.10) | 0.01 (0.09) | 0.01 (0.09) | 0.01 (0.10) |
| $w_{8}$ | 0.02 (0.11) | 0.01 (0.10) | 0.01 (0.10) | 0.01 (0.10) | 0.01 (0.10) |
| $w_{9}$ | 0.04 (0.10) | 0.01 (0.09) | 0.01 (0.08) | 0.02 (0.08) | 0.01 (0.08) |
| $w_{10}$ | 0.02 (0.09) | 0.01 (0.09) | 0.01 (0.08) | 0.01 (0.08) | 0.01 (0.09) |
| $\alpha_{0}$ | 0.45 (0.44) | 0.06 (0.16) | 0.00 (0.18) | 0.01 (0.17) | 0.01 (0.17) |
| $\alpha_{1}$ | -0.04 (0.33) | 0.04 (0.18) | 0.02 (0.18) | 0.02 (0.18) | 0.03 (0.18) |
| $\alpha_{2}$ | -0.09 (0.40) | 0.01 (0.27) | -0.02 (0.26) | -0.01 (0.27) | -0.01 (0.27) |
| $R^{2}$ | 0.83 | 0.88 | 0.90 | 0.90 | 0.90 |
| **Scenario 2B** | Complete case | LOD/$\sqrt{2}$ | MI | Truncated MI | F-AFT |
|  | Moderate correlation ($\sigma=1/2$) | | | | |
| $\psi$ | -0.33 (0.34) | 0.09 (0.22) | -0.10 (0.19) | -0.12 (0.19) | -0.12 (0.19) |
| $w_{1}$ | -0.14 (0.04) | -0.11 (0.04) | -0.15 (0.02) | -0.15 (0.02) | -0.16 (0.02) |
| $w_{2}$ | -0.20 (0.05) | -0.22 (0.03) | -0.21 (0.03) | -0.21 (0.03) | -0.21 (0.03) |
| $w_{3}$ | 0.04 (0.07) | 0.04 (0.06) | 0.01 (0.04) | 0.02 (0.05) | 0.03 (0.05) |
| $w_{4}$ | -0.02 (0.08) | 0.00 (0.06) | 0.02 (0.07) | 0.01 (0.07) | 0.01 (0.07) |
| $w_{5}$ | -0.01 (0.07) | 0.01 (0.08) | -0.01 (0.06) | 0.01 (0.07) | 0.00 (0.07) |
| $w_{6}$ | 0.01 (0.07) | 0.00 (0.05) | 0.01 (0.05) | 0.00 (0.05) | 0.01 (0.05) |
| $w_{7}$ | 0.01 (0.06) | 0.01 (0.05) | 0.00 (0.04) | 0.00 (0.04) | 0.00 (0.04) |
| $w_{8}$ | 0.02 (0.06) | 0.00 (0.04) | 0.00 (0.04) | 0.00 (0.04) | 0.00 (0.04) |
| $w_{9}$ | 0.02 (0.05) | 0.01 (0.04) | 0.01 (0.04) | 0.00 (0.04) | 0.00 (0.04) |
| $w_{10}$ | 0.27 (0.10) | 0.26 (0.06) | 0.31 (0.06) | 0.31 (0.06) | 0.31 (0.07) |
| $\alpha_{0}$ | 1.12 (0.46) | 0.35 (0.22) | 0.17 (0.23) | 0.18 (0.23) | 0.21 (0.22) |
| $\alpha_{1}$ | 0.23 (0.22) | 0.23 (0.11) | 0.24 (0.11) | 0.24 (0.11) | 0.22 (0.12) |
| $\alpha_{2}$ | -0.02 (0.34) | 0.02 (0.20) | 0.01 (0.20) | 0.01 (0.20) | -0.01 (0.20) |
| $R^{2}$ | 0.45 | 0.57 | 0.50 | 0.50 | 0.52 |
|  | High correlation ($\sigma=1/8$) | | | | |
| $\psi$ | -0.01 (0.34) | 0.04 (0.29) | 0.03 (0.25) | 0.03 (0.26) | 0.01 (0.26) |
| $w_{1}$ | -0.06 (0.04) | -0.06 (0.05) | -0.08 (0.01) | -0.08 (0.01) | -0.08 (0.01) |
| $w_{2}$ | -0.06 (0.08) | -0.07 (0.07) | -0.07 (0.07) | -0.07 (0.07) | -0.07 (0.08) |
| $w_{3}$ | 0.01 (0.10) | 0.01 (0.11) | 0.01 (0.09) | 0.01 (0.10) | 0.01 (0.10) |
| $w_{4}$ | -0.03 (0.09) | -0.01 (0.09) | 0.00 (0.10) | 0.00 (0.10) | 0.00 (0.11) |
| $w_{5}$ | -0.02 (0.09) | 0.01 (0.11) | 0.00 (0.10) | 0.00 (0.10) | 0.00 (0.10) |
| $w_{6}$ | -0.01 (0.08) | -0.01 (0.08) | 0.00 (0.09) | 0.00 (0.09) | 0.00 (0.09) |
| $w_{7}$ | 0.01 (0.09) | 0.01 (0.09) | 0.01 (0.08) | 0.00 (0.08) | 0.00 (0.09) |
| $w_{8}$ | 0.01 (0.09) | -0.01 (0.08) | 0.00 (0.09) | 0.00 (0.09) | 0.00 (0.09) |
| $w_{9}$ | 0.03 (0.09) | 0.01 (0.08) | 0.01 (0.07) | 0.01 (0.07) | 0.00 (0.07) |
| $w_{10}$ | 0.12 (0.12) | 0.12 (0.12) | 0.13 (0.13) | 0.14 (0.13) | 0.14 (0.13) |
| $\alpha_{0}$ | 0.45 (0.44) | 0.06 (0.16) | 0.00 (0.18) | 0.01 (0.18) | 0.01 (0.17) |
| $\alpha_{1}$ | -0.04 (0.33) | 0.04 (0.18) | 0.02 (0.18) | 0.02 (0.18) | 0.02 (0.18) |
| $\alpha_{2}$ | -0.09 (0.40) | 0.01 (0.27) | -0.02 (0.26) | -0.02 (0.27) | -0.01 (0.27) |
| $R^{2}$ | 0.82 | 0.88 | 0.89 | 0.89 | 0.89 |
| **Scenario 3** | Complete case | LOD/$\sqrt{2}$ | MI | Truncated MI | F-AFT |
|  | Moderate correlation ($\sigma=1/2$) | | | | |
| $\psi$ | -0.46 (0.35) | 0.16 (0.23) | -0.14 (0.20) | 0.03 (0.19) | 0.01 (0.20) |
| $w_{1}$ | 0.00 (0.10) | -0.01 (0.06) | 0.05 (0.07) | -0.01 (0.06) | 0.00 (0.06) |
| $w_{2}$ | -0.11 (0.10) | -0.01 (0.08) | -0.10 (0.07) | 0.01 (0.07) | 0.00 (0.07) |
| $w_{3}$ | 0.03 (0.06) | 0.01 (0.04) | 0.01 (0.04) | 0.00 (0.04) | 0.00 (0.04) |
| $w_{4}$ | 0.00 (0.08) | 0.00 (0.06) | 0.02 (0.07) | 0.00 (0.06) | 0.00 (0.06) |
| $w_{5}$ | 0.00 (0.08) | 0.00 (0.06) | -0.01 (0.05) | 0.00 (0.06) | 0.00 (0.06) |
| $w_{6}$ | 0.01 (0.06) | 0.00 (0.04) | 0.01 (0.05) | 0.00 (0.04) | 0.00 (0.05) |
| $w_{7}$ | 0.02 (0.06) | 0.01 (0.04) | 0.00 (0.04) | 0.00 (0.04) | 0.00 (0.04) |
| $w_{8}$ | 0.02 (0.06) | 0.00 (0.03) | 0.01 (0.04) | 0.00 (0.04) | 0.00 (0.04) |
| $w_{9}$ | 0.02 (0.06) | 0.00 (0.04) | 0.01 (0.04) | 0.00 (0.03) | 0.00 (0.03) |
| $w_{10}$ | 0.02 (0.05) | 0.00 (0.03) | 0.00 (0.03) | 0.00 (0.03) | 0.00 (0.03) |
| $\alpha_{0}$ | 1.76 (0.52) | 0.38 (0.23) | 0.06 (0.26) | -0.02 (0.24) | 0.03 (0.24) |
| $\alpha_{1}$ | 0.01 (0.26) | 0.09 (0.14) | 0.16 (0.14) | -0.02 (0.14) | -0.03 (0.14) |
| $\alpha_{2}$ | -0.03 (0.36) | 0.01 (0.20) | 0.01 (0.20) | 0.00 (0.20) | -0.02 (0.20) |
| $R^{2}$ | 0.69 | 0.85 | 0.75 | 0.83 | 0.84 |
|  | High correlation ($\sigma=1/8$) | | | | |
| $\psi$ | -0.54 (0.44) | 0.20 (0.24) | -0.14 (0.21) | 0.05 (0.19) | 0.01 (0.20) |
| $w_{1}$ | -0.03 (0.11) | -0.02 (0.06) | 0.05 (0.07) | -0.01 (0.06) | 0.00 (0.06) |
| $w_{2}$ | -0.12 (0.11) | 0.01 (0.07) | -0.10 (0.07) | 0.02 (0.07) | 0.00 (0.07) |
| $w_{3}$ | 0.04 (0.07) | 0.01 (0.04) | 0.01 (0.04) | 0.00 (0.04) | 0.00 (0.04) |
| $w_{4}$ | -0.01 (0.09) | 0.00 (0.06) | 0.02 (0.07) | 0.00 (0.06) | 0.01 (0.06) |
| $w_{5}$ | -0.01 (0.07) | 0.00 (0.06) | -0.01 (0.05) | 0.00 (0.06) | -0.01 (0.06) |
| $w_{6}$ | 0.02 (0.07) | 0.00 (0.04) | 0.01 (0.05) | 0.00 (0.04) | 0.00 (0.05) |
| $w_{7}$ | 0.02 (0.06) | 0.00 (0.04) | 0.00 (0.04) | 0.00 (0.04) | -0.01 (0.04) |
| $w_{8}$ | 0.03 (0.07) | 0.00 (0.03) | 0.01 (0.04) | 0.00 (0.04) | 0.00 (0.04) |
| $w_{9}$ | 0.03 (0.06) | 0.00 (0.04) | 0.01 (0.03) | 0.00 (0.03) | 0.00 (0.03) |
| $w_{10}$ | 0.03 (0.06) | 0.00 (0.03) | 0.00 (0.03) | 0.00 (0.03) | 0.00 (0.03) |
| $\alpha_{0}$ | 2.01 (0.69) | 0.37 (0.23) | 0.04 (0.26) | -0.04 (0.24) | 0.06 (0.24) |
| $\alpha_{1}$ | 0.05 (0.33) | 0.07 (0.14) | 0.16 (0.14) | -0.04 (0.14) | -0.04 (0.15) |
| $\alpha_{2}$ | -0.03 (0.44) | 0.01 (0.20) | 0.01 (0.21) | 0.00 (0.20) | -0.05 (0.20) |
| $R^{2}$ | 0.66 | 0.86 | 0.74 | 0.83 | 0.84 |
| **Scenario 4** | Complete case | LOD/$\sqrt{2}$ | MI | Truncated MI | F-AFT |
|  | Moderate correlation ($\sigma=1/2$) | | | | |
| $\psi$ | -0.35 (0.34) | 0.18 (0.22) | -0.08 (0.18) | 0.02 (0.17) | 0.00 (0.18) |
| $w_{1}$ | -0.02 (0.11) | -0.02 (0.06) | 0.03 (0.07) | 0.00 (0.07) | 0.00 (0.07) |
| $w_{2}$ | -0.06 (0.10) | 0.01 (0.08) | -0.07 (0.07) | 0.01 (0.07) | 0.00 (0.07) |
| $w_{3}$ | 0.03 (0.06) | 0.01 (0.04) | 0.00 (0.04) | 0.00 (0.04) | 0.00 (0.04) |
| $w_{4}$ | -0.02 (0.08) | -0.01 (0.06) | 0.01 (0.07) | 0.00 (0.06) | 0.00 (0.07) |
| $w_{5}$ | -0.01 (0.08) | 0.00 (0.07) | -0.01 (0.06) | 0.00 (0.06) | 0.00 (0.06) |
| $w_{6}$ | 0.01 (0.07) | 0.00 (0.05) | 0.01 (0.05) | 0.00 (0.05) | 0.00 (0.05) |
| $w_{7}$ | 0.02 (0.06) | 0.00 (0.04) | 0.00 (0.04) | 0.00 (0.04) | 0.00 (0.04) |
| $w_{8}$ | 0.02 (0.06) | 0.00 (0.03) | 0.00 (0.04) | 0.00 (0.04) | 0.00 (0.04) |
| $w_{9}$ | 0.02 (0.05) | 0.00 (0.04) | 0.00 (0.04) | 0.00 (0.04) | 0.00 (0.04) |
| $w_{10}$ | 0.02 (0.05) | 0.00 (0.03) | 0.00 (0.03) | 0.00 (0.03) | 0.00 (0.03) |
| $\alpha_{0}$ | 1.46 (0.53) | 0.32 (0.21) | 0.01 (0.23) | -0.01 (0.22) | 0.03 (0.22) |
| $\alpha_{1}$ | 0.02 (0.25) | 0.06 (0.12) | 0.10 (0.12) | -0.01 (0.12) | -0.02 (0.13) |
| $\alpha_{2}$ | -0.05 (0.36) | 0.01 (0.19) | 0.01 (0.19) | 0.00 (0.18) | -0.02 (0.19) |
| $R^{2}$ | 0.72 | 0.86 | 0.78 | 0.84 | 0.85 |
|  | High correlation ($\sigma=1/8$) | | | | |
| $\psi$ | -0.45 (0.42) | 0.20 (0.22) | -0.08 (0.18) | 0.03 (0.18) | 0.00 (0.18) |
| $w_{1}$ | -0.05 (0.11) | -0.03 (0.06) | 0.04 (0.07) | -0.01 (0.06) | 0.00 (0.07) |
| $w_{2}$ | -0.08 (0.10) | 0.02 (0.08) | -0.07 (0.06) | 0.02 (0.07) | 0.00 (0.07) |
| $w_{3}$ | 0.04 (0.07) | 0.01 (0.04) | 0.00 (0.04) | 0.00 (0.04) | 0.00 (0.04) |
| $w_{4}$ | -0.02 (0.08) | -0.01 (0.06) | 0.01 (0.07) | 0.00 (0.06) | 0.01 (0.06) |
| $w_{5}$ | -0.01 (0.08) | 0.00 (0.07) | -0.01 (0.06) | 0.00 (0.06) | -0.01 (0.06) |
| $w_{6}$ | 0.02 (0.07) | 0.00 (0.04) | 0.01 (0.05) | 0.00 (0.05) | 0.00 (0.05) |
| $w_{7}$ | 0.02 (0.07) | 0.00 (0.04) | 0.00 (0.04) | 0.00 (0.04) | -0.01 (0.04) |
| $w_{8}$ | 0.02 (0.06) | 0.00 (0.03) | 0.00 (0.04) | 0.00 (0.04) | 0.00 (0.04) |
| $w_{9}$ | 0.03 (0.07) | 0.00 (0.04) | 0.01 (0.04) | 0.00 (0.04) | 0.00 (0.04) |
| $w_{10}$ | 0.03 (0.06) | 0.00 (0.03) | 0.00 (0.03) | 0.00 (0.03) | 0.00 (0.03) |
| $\alpha_{0}$ | 1.75 (0.70) | 0.32 (0.21) | 0.00 (0.24) | -0.02 (0.22) | 0.06 (0.22) |
| $\alpha_{1}$ | 0.05 (0.32) | 0.05 (0.12) | 0.12 (0.12) | -0.02 (0.13) | -0.03 (0.14) |
| $\alpha_{2}$ | -0.03 (0.46) | 0.01 (0.19) | 0.01 (0.19) | 0.00 (0.18) | -0.04 (0.19) |
| $R^{2}$ | 0.69 | 0.87 | 0.76 | 0.84 | 0.85 |

**Abbreviations**: Imputation by LOD/$\sqrt{2}$ (LOD/$\sqrt{2}$); conventional multiple imputation (MI); truncated multiple imputation (Truncated MI); imputation by estimates using the AFT model (F-AFT).

*Note*: $\psi$ is the total effect of the mixture; $w_{j}$ is the weight for each quantized exposure $\bar{Z}_{j} (j=1,\ldots,10)$; $\alpha_{k}$ is a coefficient for intercept, $X_{1}$ and $X_{2}$ $\left( k=0,1,2 \right)$; and $R^{2}$ was calculated by regression $\hat{h}$ from each LOD accommodation on $\hat{h}$ with the full dataset.

**Table S3. Posterior inclusion probability (PIP) of mixture exposures from BKMR using NHANES 2001-2002 data.**

|  | Complete case | LOD/$\sqrt{2}$ | MI | Truncated MI | F-AFT |
| --- | --- | --- | --- | --- | --- |
|  | Group PIP | | | | |
| Non-Dioxin-like | 0.318 | 0.302 | 0.344 | 0.374 | 0.640 |
| Non-Ortho | 0.434 | 0.968 | 0.331 | 0.795 | 0.788 |
| mPFDs | 0.868 | 0.520 | 0.972 | 0.730 | 0.870 |
|  | Conditional PIP within Non-Dioxin-like PCB group | | | | |
| PCB74 | 0.119 | 0.132 | 0.068 | 0.110 | 0.228 |
| PCB99 | 0.000 | 0.086 | 0.040 | 0.262 | 0.056 |
| PCB138 | 0.226 | 0.040 | 0.042 | 0.078 | 0.091 |
| PCB153 | 0.082 | 0.113 | 0.057 | 0.097 | 0.078 |
| PCB170 | 0.340 | 0.325 | 0.190 | 0.257 | 0.222 |
| PCB180 | 0.189 | 0.126 | 0.396 | 0.054 | 0.078 |
| PCB187 | 0.006 | 0.020 | 0.049 | 0.074 | 0.119 |
| PCB194 | 0.038 | 0.159 | 0.159 | 0.067 | 0.128 |
|  | Conditional PIP within mPFDs group | | | | |
| PCB118 | 0.023 | 0.095 | 0.247 | 0.062 | 0.117 |
| 1,2,3,6,7,8-hxcdd | 0.161 | 0.000 | 0.057 | 0.019 | 0.023 |
| 1,2,3,4,6,7,8-hpcdd | 0.000 | 0.000 | 0.029 | 0.007 | 0.000 |
| 1,2,3,4,6,7,8,9-ocdd | 0.060 | 0.000 | 0.024 | 0.011 | 0.013 |
| 2,3,4,7,8-pncdf | 0.281 | 0.884 | 0.285 | 0.793 | 0.673 |
| 1,2,3,4,7,8-hxcdf | 0.037 | 0.000 | 0.169 | 0.029 | 0.071 |
| 1,2,3,6,7,8-hxcdf | 0.350 | 0.021 | 0.122 | 0.027 | 0.005 |
| 1,2,3,4,6,7,8-hxcdf | 0.088 | 0.000 | 0.067 | 0.052 | 0.099 |
|  | Conditional PIP within Non-Ortho PCB group | | | | |
| PCB169 | 0.896 | 0.508 | 0.934 | 0.515 | 0.439 |
| PCB126 | 0.104 | 0.492 | 0.066 | 0.485 | 0.561 |

**Abbreviations**: Imputation by LOD/$\sqrt{2}$ (LOD/$\sqrt{2}$); conventional multiple imputation (MI); truncated multiple imputation (Truncated MI); imputation by estimates using the AFT model (F-AFT).

**Table S4. Simulation results of linear regression with different LOD accommodation approaches under Scenario 1.**

| **Bias (SE)** | Complete case | LOD/$\sqrt{2}$ | MI | Truncated MI | F-AFT |
| --- | --- | --- | --- | --- | --- |
|  | Moderate correlation ($\sigma=1/2$) | | | | |
| $\beta_{1}$ | 0.002 (0.261) | 0.035 (0.141) | 0.061 (0.144) | 0.004 (0.143) | 0.028 (0.141) |
| $\beta_{2}$ | -0.005 (0.297) | -0.030 (0.140) | 0.018 (0.204) | 0.006 (0.145) | 0.020 (0.147) |
| $\beta_{3}$ | -0.012 (0.336) | 0.008 (0.148) | 0.021 (0.241) | -0.006 (0.154) | -0.013 (0.161) |
| $\beta_{4}$ | -0.002 (0.448) | 0.034 (0.210) | 0.040 (0.231) | -0.002 (0.220) | 0.035 (0.211) |
| $\beta_{5}$ | -0.021 (0.486) | -0.056 (0.204) | -0.001 (0.323) | 0.006 (0.240) | -0.026 (0.226) |
| $\beta_{6}$ | 0.022 (0.420) | 0.037 (0.214) | 0.051 (0.224) | 0.000 (0.224) | 0.039 (0.213) |
| $\beta_{7}$ | -0.025 (0.391) | -0.021 (0.168) | -0.008 (0.259) | -0.005 (0.184) | -0.010 (0.185) |
| $\beta_{8}$ | 0.022 (0.333) | 0.013 (0.172) | 0.020 (0.176) | -0.006 (0.178) | 0.005 (0.172) |
| $\beta_{9}$ | -0.013 (0.349) | -0.021 (0.171) | -0.005 (0.237) | 0.008 (0.185) | -0.001 (0.182) |
| $\beta_{10}$ | 0.001 (0.312) | 0.018 (0.162) | 0.025 (0.169) | -0.001 (0.167) | 0.009 (0.161) |
| $\alpha_{0}$ | 0.015 (0.559) | -0.085 (0.155) | -0.504 (0.294) | -0.006 (0.149) | -0.075 (0.158) |
| $\alpha_{1}$ | 0.015 (0.226) | 0.066 (0.108) | 0.108 (0.120) | 0.001 (0.119) | -0.001 (0.118) |
| $\alpha_{2}$ | -0.008 (0.291) | 0.002 (0.153) | 0.006 (0.158) | -0.002 (0.154) | -0.008 (0.154) |
|  | High correlation ($\sigma=1/8$) | | | | |
| $\beta_{1}$ | 0.053 (0.868) | 0.123 (0.546) | 0.041 (0.581) | 0.019 (0.573) | 0.064 (0.555) |
| $\beta_{2}$ | -0.038 (0.845) | -0.404 (0.393) | -0.017 (0.697) | -0.006 (0.628) | -0.026 (0.610) |
| $\beta_{3}$ | -0.030 (0.906) | -0.012 (0.396) | -0.051 (0.762) | -0.018 (0.628) | -0.026 (0.616) |
| $\beta_{4}$ | -0.019 (1.395) | 0.074 (0.813) | 0.024 (0.904) | -0.008 (0.885) | 0.039 (0.835) |
| $\beta_{5}$ | 0.049 (1.435) | -0.234 (0.467) | 0.003 (1.154) | 0.014 (0.988) | -0.052 (0.839) |
| $\beta_{6}$ | 0.005 (1.335) | 0.093 (0.825) | 0.039 (0.896) | 0.012 (0.893) | 0.060 (0.837) |
| $\beta_{7}$ | -0.023 (1.071) | -0.113 (0.457) | -0.035 (0.868) | -0.016 (0.765) | -0.037 (0.732) |
| $\beta_{8}$ | 0.028 (1.061) | 0.070 (0.658) | -0.010 (0.708) | -0.025 (0.709) | -0.002 (0.672) |
| $\beta_{9}$ | 0.008 (1.025) | -0.180 (0.446) | 0.013 (0.844) | 0.020 (0.802) | 0.005 (0.748) |
| $\beta_{10}$ | -0.012 (1.016) | 0.090 (0.615) | 0.018 (0.683) | 0.002 (0.671) | 0.021 (0.635) |
| $\alpha_{0}$ | -0.014 (0.752) | 0.201 (0.334) | -0.081 (0.519) | 0.000 (0.396) | -0.010 (0.401) |
| $\alpha_{1}$ | 0.015 (0.598) | 0.253 (0.274) | 0.037 (0.435) | 0.005 (0.440) | 0.003 (0.435) |
| $\alpha_{2}$ | -0.021 (0.485) | 0.006 (0.330) | 0.009 (0.333) | 0.003 (0.331) | -0.012 (0.332) |
| **MSE** | Complete case | LOD/$\sqrt{2}$ | MI | Truncated MI | F-AFT |
|  | Moderate correlation ($\sigma=1/2$) | | | | |
| $\beta_{1}$ | 0.068 | 0.021 | 0.024 | 0.020 | 0.021 |
| $\beta_{2}$ | 0.088 | 0.020 | 0.042 | 0.021 | 0.022 |
| $\beta_{3}$ | 0.113 | 0.022 | 0.058 | 0.024 | 0.026 |
| $\beta_{4}$ | 0.201 | 0.045 | 0.055 | 0.048 | 0.046 |
| $\beta_{5}$ | 0.236 | 0.045 | 0.104 | 0.057 | 0.052 |
| $\beta_{6}$ | 0.177 | 0.047 | 0.053 | 0.050 | 0.047 |
| $\beta_{7}$ | 0.153 | 0.029 | 0.067 | 0.034 | 0.034 |
| $\beta_{8}$ | 0.111 | 0.030 | 0.031 | 0.032 | 0.030 |
| $\beta_{9}$ | 0.122 | 0.030 | 0.056 | 0.034 | 0.033 |
| $\beta_{10}$ | 0.097 | 0.026 | 0.029 | 0.028 | 0.026 |
| $\alpha_{0}$ | 0.313 | 0.031 | 0.340 | 0.022 | 0.031 |
| $\alpha_{1}$ | 0.051 | 0.016 | 0.026 | 0.014 | 0.014 |
| $\alpha_{2}$ | 0.084 | 0.023 | 0.025 | 0.024 | 0.024 |
|  | High correlation ($\sigma=1/8$) | | | | |
| $\beta_{1}$ | 0.755 | 0.313 | 0.338 | 0.328 | 0.312 |
| $\beta_{2}$ | 0.715 | 0.317 | 0.485 | 0.394 | 0.373 |
| $\beta_{3}$ | 0.820 | 0.157 | 0.582 | 0.394 | 0.379 |
| $\beta_{4}$ | 1.944 | 0.666 | 0.817 | 0.783 | 0.699 |
| $\beta_{5}$ | 2.060 | 0.273 | 1.331 | 0.976 | 0.706 |
| $\beta_{6}$ | 1.781 | 0.688 | 0.803 | 0.797 | 0.703 |
| $\beta_{7}$ | 1.146 | 0.222 | 0.754 | 0.586 | 0.537 |
| $\beta_{8}$ | 1.126 | 0.437 | 0.501 | 0.503 | 0.452 |
| $\beta_{9}$ | 1.050 | 0.231 | 0.712 | 0.643 | 0.558 |
| $\beta_{10}$ | 1.031 | 0.386 | 0.466 | 0.450 | 0.404 |
| $\alpha_{0}$ | 0.566 | 0.152 | 0.276 | 0.156 | 0.161 |
| $\alpha_{1}$ | 0.358 | 0.139 | 0.191 | 0.193 | 0.189 |
| $\alpha_{2}$ | 0.235 | 0.109 | 0.111 | 0.109 | 0.111 |
| **CP** | Complete case | LOD/$\sqrt{2}$ | MI | Truncated MI | F-AFT |
|  | Moderate correlation ($\sigma=1/2$) | | | | |
| $\beta_{1}$ | 0.958 | 0.937 | 0.921 | 0.943 | 0.940 |
| $\beta_{2}$ | 0.954 | 0.950 | 0.947 | 0.941 | 0.942 |
| $\beta_{3}$ | 0.947 | 0.936 | 0.925 | 0.938 | 0.941 |
| $\beta_{4}$ | 0.933 | 0.942 | 0.939 | 0.945 | 0.938 |
| $\beta_{5}$ | 0.942 | 0.941 | 0.937 | 0.940 | 0.945 |
| $\beta_{6}$ | 0.949 | 0.939 | 0.945 | 0.940 | 0.942 |
| $\beta_{7}$ | 0.931 | 0.945 | 0.937 | 0.945 | 0.946 |
| $\beta_{8}$ | 0.946 | 0.926 | 0.932 | 0.931 | 0.927 |
| $\beta_{9}$ | 0.953 | 0.944 | 0.944 | 0.943 | 0.942 |
| $\beta_{10}$ | 0.957 | 0.946 | 0.947 | 0.947 | 0.949 |
| $\alpha_{0}$ | 0.951 | 0.911 | 0.550 | 0.944 | 0.912 |
| $\alpha_{1}$ | 0.946 | 0.913 | 0.848 | 0.951 | 0.950 |
| $\alpha_{2}$ | 0.944 | 0.937 | 0.936 | 0.940 | 0.935 |
|  | High correlation ($\sigma=1/8$) | | | | |
| $\beta_{1}$ | 0.937 | 0.943 | 0.938 | 0.943 | 0.942 |
| $\beta_{2}$ | 0.956 | 0.831 | 0.939 | 0.939 | 0.948 |
| $\beta_{3}$ | 0.946 | 0.957 | 0.929 | 0.936 | 0.951 |
| $\beta_{4}$ | 0.931 | 0.948 | 0.940 | 0.944 | 0.947 |
| $\beta_{5}$ | 0.947 | 0.914 | 0.951 | 0.944 | 0.949 |
| $\beta_{6}$ | 0.946 | 0.947 | 0.949 | 0.944 | 0.950 |
| $\beta_{7}$ | 0.938 | 0.934 | 0.941 | 0.941 | 0.949 |
| $\beta_{8}$ | 0.938 | 0.931 | 0.939 | 0.926 | 0.926 |
| $\beta_{9}$ | 0.949 | 0.916 | 0.936 | 0.925 | 0.948 |
| $\beta_{10}$ | 0.943 | 0.953 | 0.951 | 0.953 | 0.947 |
| $\alpha_{0}$ | 0.947 | 0.905 | 0.942 | 0.946 | 0.946 |
| $\alpha_{1}$ | 0.948 | 0.827 | 0.943 | 0.940 | 0.958 |
| $\alpha_{2}$ | 0.942 | 0.947 | 0.942 | 0.950 | 0.945 |

**Abbreviations**: Imputation by LOD/$\sqrt{2}$ (LOD/$\sqrt{2}$); conventional multiple imputation (MI); truncated multiple imputation (Truncated MI); imputation by estimates using the AFT model (F-AFT); mean squared error (MSE); coverage probability of the 95% confidence interval (CP).

*Note*: we compared the results of each LOD accommodation approach with true underlying model (i.e., true parameters).

**Table S5. Group lasso: bias (SE) of each LOD accommodation approach compared to using full dataset under Scenario 1.**

| **Group Lasso** | Complete case | LOD/$\sqrt{2}$ | MI | Truncated MI | F-AFT |
| --- | --- | --- | --- | --- | --- |
|  | Moderate correlation ($\sigma=1/2$) | | | | |
| $\beta_{1}$ | 0.319 (0.146) | -0.073 (0.154) | -0.446 (0.260) | -0.007 (0.147) | -0.058 (0.154) |
| $\beta_{2}$ | -0.101 (0.137) | 0.029 (0.139) | 0.071 (0.138) | 0.006 (0.138) | 0.026 (0.137) |
| $\beta_{3}$ | -0.110 (0.125) | -0.033 (0.129) | 0.004 (0.188) | 0.010 (0.135) | 0.008 (0.135) |
| $\beta_{4}$ | 0.011 (0.128) | 0.009 (0.134) | 0.015 (0.210) | -0.005 (0.140) | -0.004 (0.144) |
| $\beta_{5}$ | -0.102 (0.159) | 0.024 (0.160) | 0.061 (0.175) | 0.006 (0.160) | 0.033 (0.158) |
| $\beta_{6}$ | -0.110 (0.144) | -0.038 (0.140) | -0.013 (0.226) | 0.006 (0.166) | -0.027 (0.149) |
| $\beta_{7}$ | 0.018 (0.158) | 0.023 (0.152) | 0.031 (0.166) | -0.006 (0.163) | 0.029 (0.152) |
| $\beta_{8}$ | -0.042 (0.117) | -0.010 (0.116) | 0.008 (0.178) | 0.010 (0.130) | -0.007 (0.122) |
| $\beta_{9}$ | 0.002 (0.110) | 0.010 (0.114) | 0.022 (0.125) | 0.001 (0.118) | 0.005 (0.111) |
| $\beta_{10}$ | -0.011 (0.103) | -0.016 (0.106) | -0.012 (0.158) | -0.001 (0.120) | -0.001 (0.106) |
| $\alpha_{0}$ | -0.001 (0.099) | 0.010 (0.099) | 0.014 (0.114) | -0.002 (0.104) | 0.005 (0.097) |
| $\alpha_{1}$ | 0.063 (0.108) | 0.061 (0.103) | 0.101 (0.111) | -0.006 (0.110) | 0.000 (0.110) |
| $\alpha_{2}$ | 0.025 (0.148) | 0.005 (0.150) | 0.002 (0.154) | -0.004 (0.149) | -0.003 (0.150) |
|  | High correlation ($\sigma=1/8$) | | | | |
| $\beta_{1}$ | -0.161 (0.580) | -0.074 (0.505) | 0.094 (0.481) | 0.043 (0.469) | 0.024 (0.488) |
| $\beta_{2}$ | -0.159 (0.473) | -0.249 (0.264) | 0.064 (0.507) | 0.047 (0.452) | -0.046 (0.416) |
| $\beta_{3}$ | -0.015 (0.388) | -0.034 (0.245) | -0.043 (0.499) | -0.014 (0.396) | -0.013 (0.349) |
| $\beta_{4}$ | -0.092 (0.442) | -0.037 (0.342) | 0.062 (0.408) | 0.023 (0.359) | 0.016 (0.346) |
| $\beta_{5}$ | -0.085 (0.468) | -0.080 (0.237) | 0.027 (0.527) | 0.019 (0.414) | -0.029 (0.332) |
| $\beta_{6}$ | -0.041 (0.419) | -0.010 (0.270) | 0.004 (0.384) | -0.003 (0.339) | 0.023 (0.294) |
| $\beta_{7}$ | -0.019 (0.392) | -0.029 (0.215) | 0.025 (0.461) | 0.032 (0.372) | -0.006 (0.289) |
| $\beta_{8}$ | -0.001 (0.401) | 0.015 (0.302) | 0.011 (0.369) | -0.003 (0.348) | 0.006 (0.313) |
| $\beta_{9}$ | -0.010 (0.373) | -0.074 (0.231) | -0.003 (0.459) | 0.002 (0.395) | -0.006 (0.303) |
| $\beta_{10}$ | -0.014 (0.380) | 0.011 (0.252) | 0.001 (0.339) | -0.010 (0.299) | 0.010 (0.270) |
| $\alpha_{0}$ | 0.257 (0.591) | 0.193 (0.301) | -0.120 (0.429) | -0.036 (0.356) | 0.011 (0.374) |
| $\alpha_{1}$ | 0.133 (0.418) | 0.196 (0.258) | -0.035 (0.351) | -0.038 (0.354) | 0.018 (0.350) |
| $\alpha_{2}$ | 0.024 (0.324) | 0.036 (0.261) | -0.026 (0.266) | -0.017 (0.260) | -0.005 (0.263) |

**Abbreviations**: Imputation by LOD/$\sqrt{2}$ (LOD/$\sqrt{2}$); conventional multiple imputation (MI); truncated multiple imputation (Truncated MI); imputation by estimates using the AFT model (F-AFT).

**Table S6. BKMR with hierarchical variable selection: summary measures of estimated** $\boldsymbol{h}\left( \boldsymbol{Z} \right)$ **with each LOD accommodation approach compared to using full dataset under Scenario 1.**

| LOD accommodation | Moderate correlation ($\sigma=1/2$) | | | | High correlation ($\sigma=1/8$) | | | |
| --- | --- | --- | --- | --- | --- | --- | --- | --- |
|  | Intercept | Slope | $R^{2}$ | SE | Intercept | Slope | $R^{2}$ | SE |
| Complete case | 0.498 | 0.893 | 0.783 | 0.315 | 0.593 | 0.697 | 0.631 | 0.188 |
| LOD/$\sqrt{2}$ | 0.113 | 0.942 | 0.934 | 0.189 | 0.269 | 0.858 | 0.847 | 0.146 |
| MI | 0.133 | 0.935 | 0.891 | 0.207 | -0.263 | 1.155 | 0.874 | 0.149 |
| Truncated MI | 0.028 | 0.996 | 0.939 | 0.204 | -0.288 | 1.162 | 0.914 | 0.149 |
| F-AFT | 0.098 | 0.957 | 0.940 | 0.188 | 0.049 | 0.979 | 0.907 | 0.142 |

**Abbreviations**: Imputation by LOD/$\sqrt{2}$ (LOD/$\sqrt{2}$); conventional multiple imputation (MI); truncated multiple imputation (Truncated MI); imputation by estimates using the AFT model (F-AFT).

*Note*: Summary measures were obtained by regressing the estimated $\hat{h}$ of each LOD-accommodation approach on $\hat{h}$ using full datasets, and reported average intercept, slope, and $R^{2}$ across simulation iterations. Zero intercept and 1 of slope indicate LOD accommodation approach does not influence BKMR results. “SE” denotes the posterior standard deviation of the $\hat{h}$.

**Table S7. quantile-based g-computation: bias (SE) of each LOD accommodation approach compared to using full dataset under Scenario 1.**

|  | Complete case | LOD/$\sqrt{2}$ | MI | Truncated MI | F-AFT |
| --- | --- | --- | --- | --- | --- |
|  | Moderate correlation ($\sigma=1/2$) | | | | |
| $\psi$ | 0.26 (0.29) | -0.15 (0.19) | 0.07 (0.17) | 0.00 (0.16) | 0.02 (0.16) |
| $w_{1}$ | 0.00 (0.08) | 0.02 (0.04) | -0.03 (0.05) | 0.00 (0.04) | 0.00 (0.04) |
| $w_{2}$ | 0.04 (0.10) | -0.01 (0.05) | 0.06 (0.05) | 0.00 (0.05) | 0.00 (0.05) |
| $w_{3}$ | 0.01 (0.24) | 0.00 (0.26) | 0.02 (0.22) | 0.00 (0.23) | 0.02 (0.25) |
| $w_{4}$ | 0.03 (0.16) | 0.00 (0.05) | -0.02 (0.06) | 0.00 (0.07) | 0.00 (0.07) |
| $w_{5}$ | 0.06 (0.19) | 0.02 (0.15) | 0.04 (0.14) | 0.00 (0.09) | 0.01 (0.10) |
| $w_{6}$ | 0.00 (0.27) | -0.04 (0.24) | -0.05 (0.22) | 0.00 (0.26) | -0.02 (0.26) |
| $w_{7}$ | 0.05 (0.26) | 0.01 (0.25) | 0.06 (0.23) | 0.00 (0.20) | 0.00 (0.22) |
| $w_{8}$ | -0.02 (0.28) | -0.01 (0.29) | -0.02 (0.26) | 0.00 (0.28) | -0.01 (0.28) |
| $w_{9}$ | -0.08 (0.32) | 0.04 (0.39) | -0.02 (0.31) | 0.00 (0.35) | 0.04 (0.38) |
| $w_{10}$ | -0.06 (0.29) | -0.03 (0.33) | -0.03 (0.30) | -0.01 (0.33) | -0.03 (0.32) |
| $\alpha_{0}$ | -1.38 (0.42) | -0.30 (0.17) | -0.01 (0.21) | 0.00 (0.19) | -0.04 (0.18) |
| $\alpha_{1}$ | 0.04 (0.22) | -0.06 (0.10) | -0.09 (0.11) | 0.00 (0.11) | 0.01 (0.11) |
| $\alpha_{2}$ | 0.02 (0.29) | -0.01 (0.16) | -0.01 (0.16) | 0.00 (0.16) | 0.02 (0.16) |
|  | High correlation ($\sigma=1/8$) | | | | |
| $\psi$ | 0.00 (0.29) | 0.00 (0.23) | 0.00 (0.22) | 0.00 (0.21) | 0.01 (0.22) |
| $w_{1}$ | 0.04 (0.18) | -0.01 (0.15) | 0.01 (0.16) | 0.00 (0.16) | -0.01 (0.16) |
| $w_{2}$ | 0.02 (0.26) | 0.03 (0.26) | 0.01 (0.22) | 0.01 (0.22) | 0.03 (0.24) |
| $w_{3}$ | -0.01 (0.21) | 0.01 (0.22) | 0.00 (0.18) | 0.00 (0.19) | 0.00 (0.21) |
| $w_{4}$ | 0.05 (0.22) | -0.01 (0.17) | 0.00 (0.18) | 0.00 (0.18) | -0.01 (0.18) |
| $w_{5}$ | 0.03 (0.22) | 0.01 (0.20) | 0.01 (0.18) | 0.00 (0.19) | 0.00 (0.19) |
| $w_{6}$ | -0.01 (0.23) | -0.02 (0.21) | -0.01 (0.21) | 0.00 (0.22) | -0.01 (0.22) |
| $w_{7}$ | 0.00 (0.25) | 0.01 (0.26) | 0.01 (0.23) | 0.00 (0.23) | 0.00 (0.25) |
| $w_{8}$ | -0.03 (0.27) | -0.01 (0.25) | -0.01 (0.24) | 0.00 (0.25) | -0.01 (0.25) |
| $w_{9}$ | -0.07 (0.31) | 0.00 (0.32) | -0.01 (0.29) | -0.01 (0.29) | 0.01 (0.31) |
| $w_{10}$ | -0.03 (0.26) | -0.02 (0.24) | -0.01 (0.25) | 0.00 (0.26) | 0.00 (0.26) |
| $\alpha_{0}$ | -0.49 (0.43) | -0.04 (0.14) | 0.00 (0.15) | 0.00 (0.15) | -0.01 (0.15) |
| $\alpha_{1}$ | 0.12 (0.37) | -0.02 (0.17) | 0.00 (0.19) | 0.00 (0.18) | -0.01 (0.19) |
| $\alpha_{2}$ | 0.06 (0.41) | 0.00 (0.27) | 0.00 (0.27) | 0.00 (0.27) | 0.01 (0.28) |

**Abbreviations**: Imputation by LOD/$\sqrt{2}$ (LOD/$\sqrt{2}$); conventional multiple imputation (MI); truncated multiple imputation (Truncated MI); imputation by estimates using the AFT model (F-AFT).

*Note*: $\psi$ is the total effect of the mixture; $w_{j}$ is the weight for each quantized exposure $\bar{Z}_{j} (j=1,\ldots,10)$; and $\alpha_{k}$ is a coefficient for intercept, $X_{1}$ and $X_{2}$ $\left( k=0,1,2 \right)$.

**Figure S1. Spearman correlations between simulated exposures.**

A. Moderate correlation setting ($\sigma=1/2$)


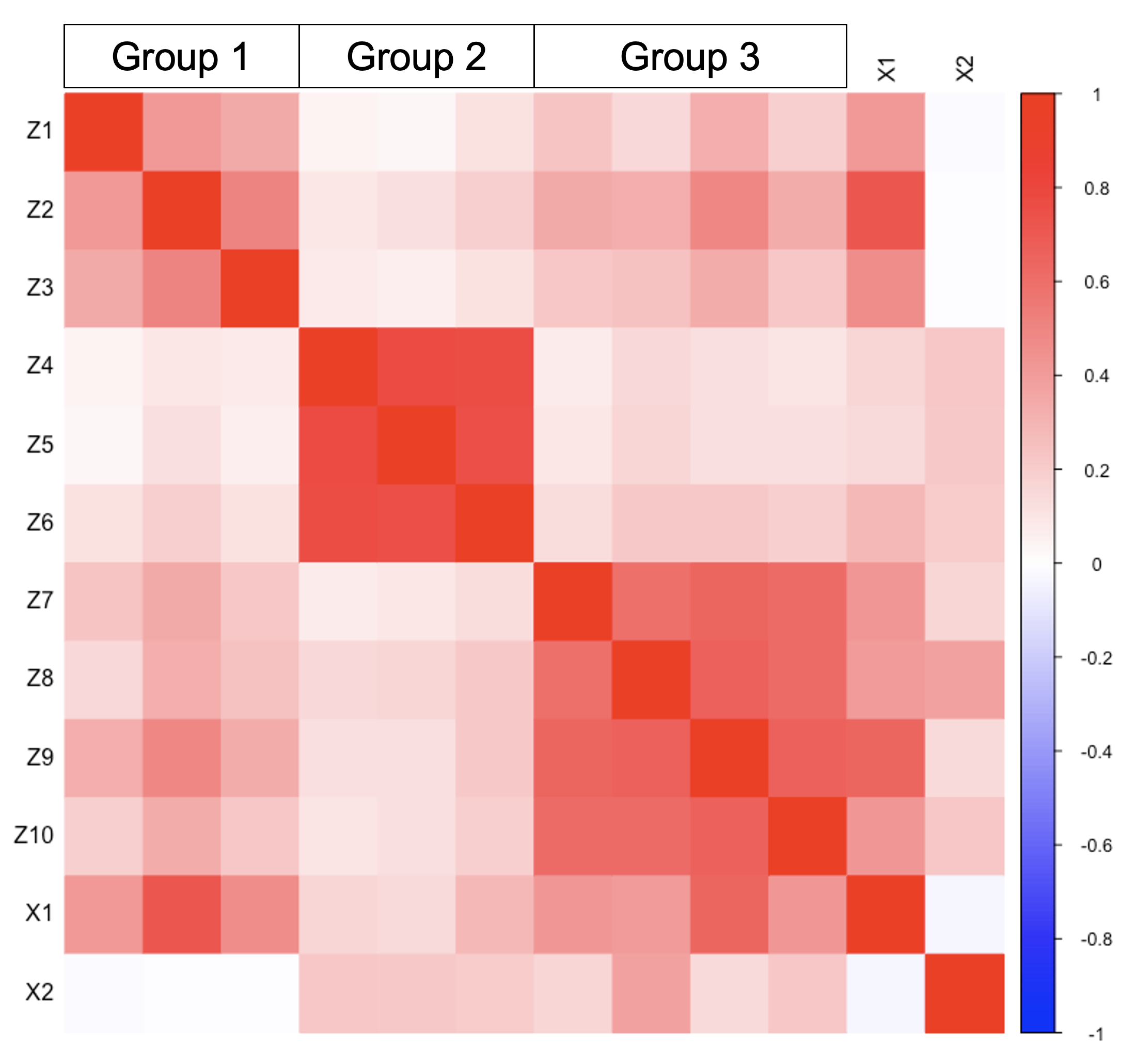


B. High correlation setting ($\sigma=1/8$)


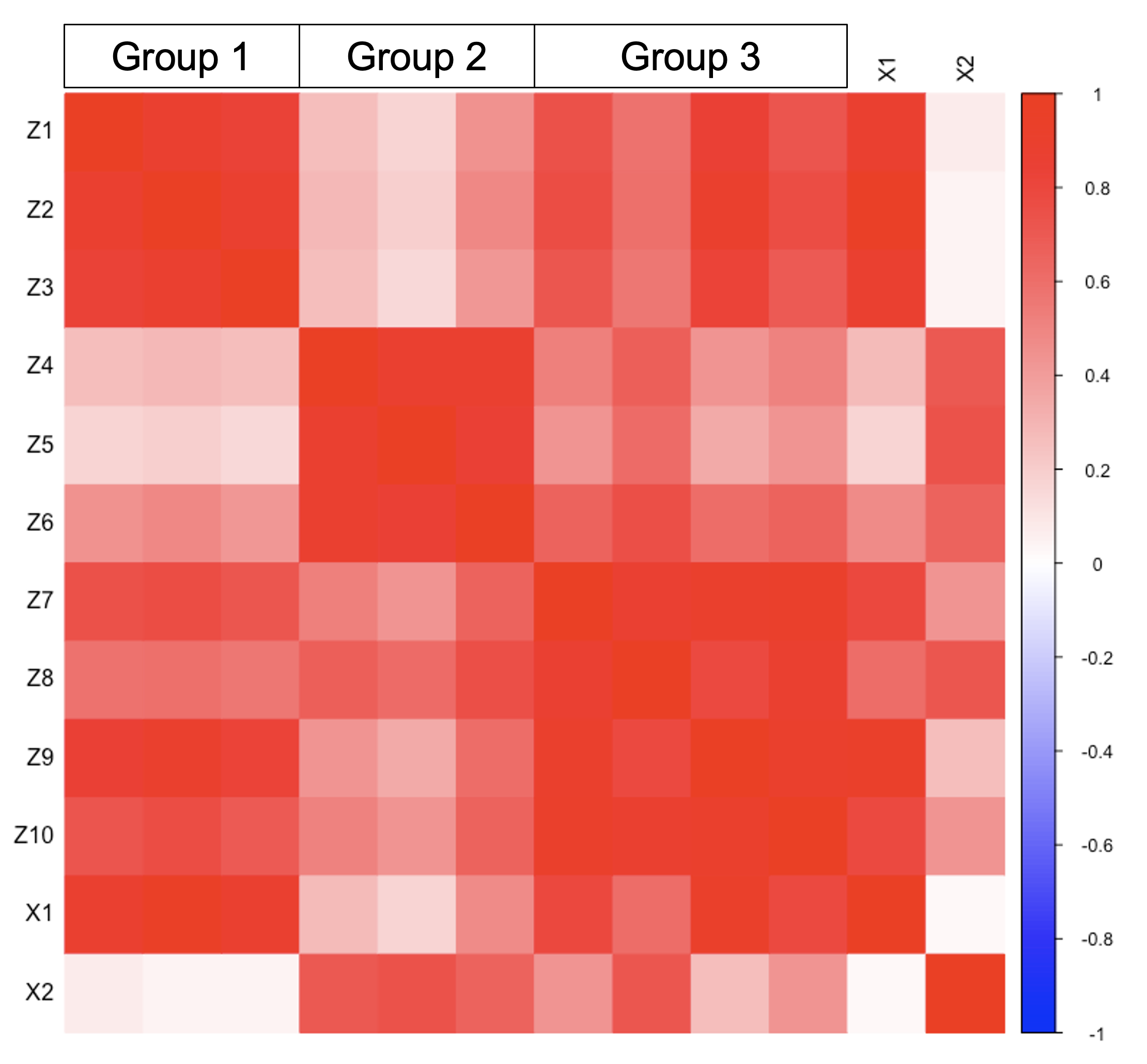


**Figure S2. Median (25%, 75%) of the posterior inclusion probability from BKMR in the simulation study.**

1. **Scenario 1 with moderate correlation setting**


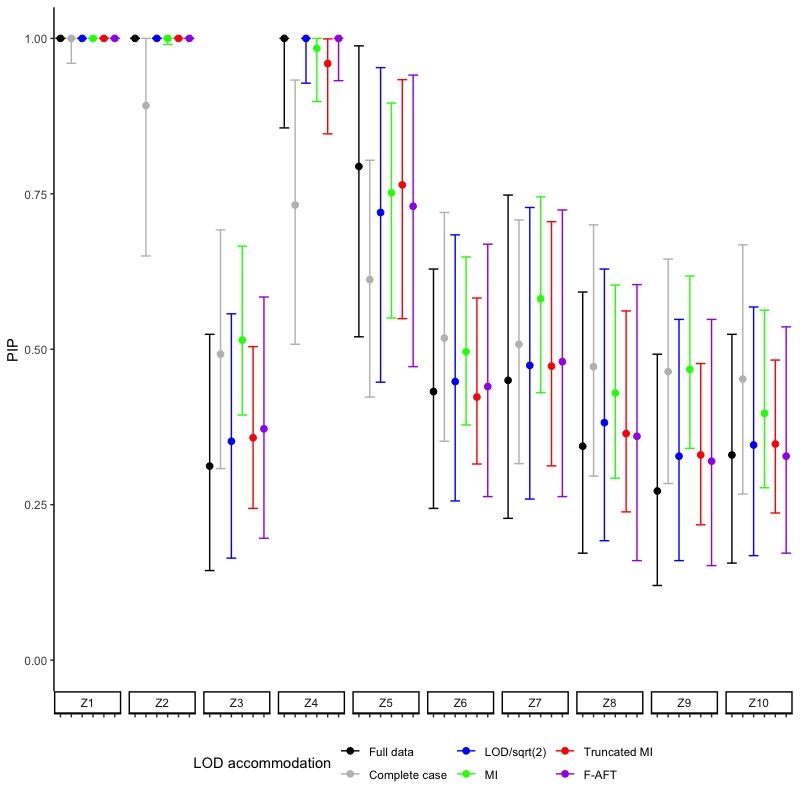


1. **Scenario 1 with high correlation setting**


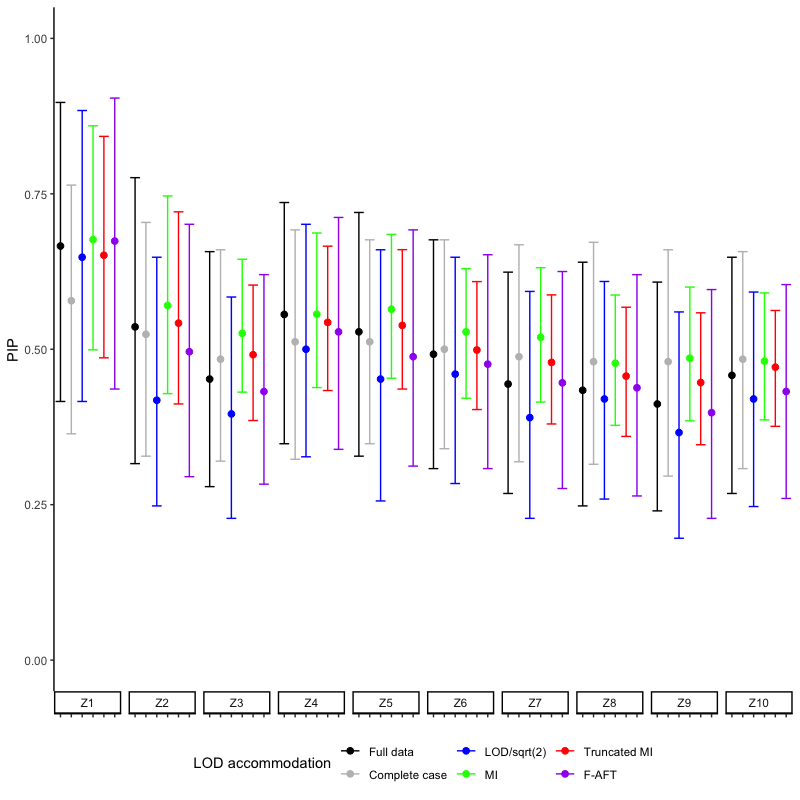


1. **Scenario 2A with moderate correlation setting**


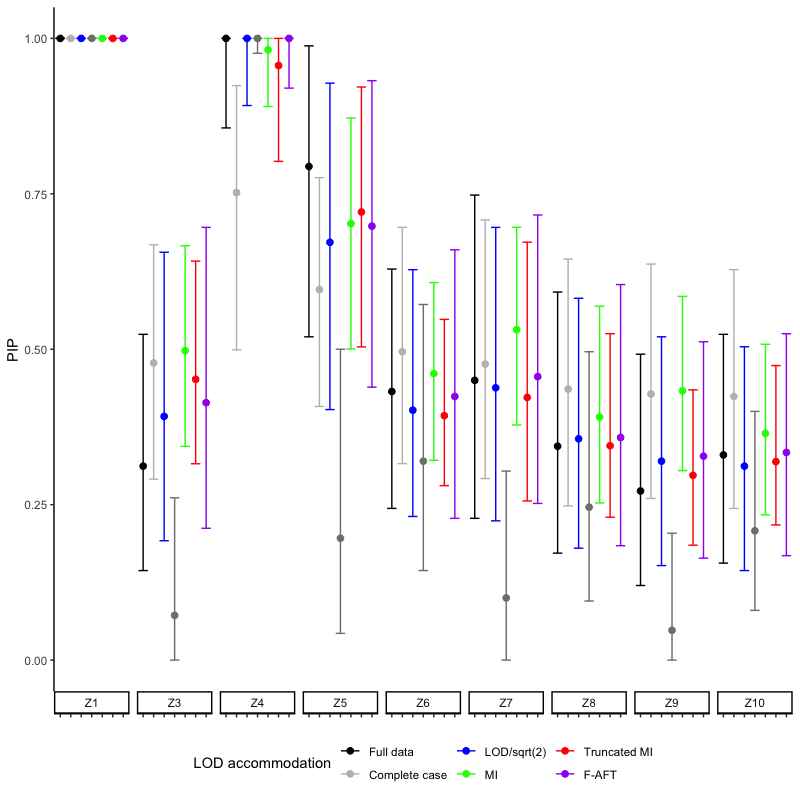


1. **Scenario 2A with high correlation setting**


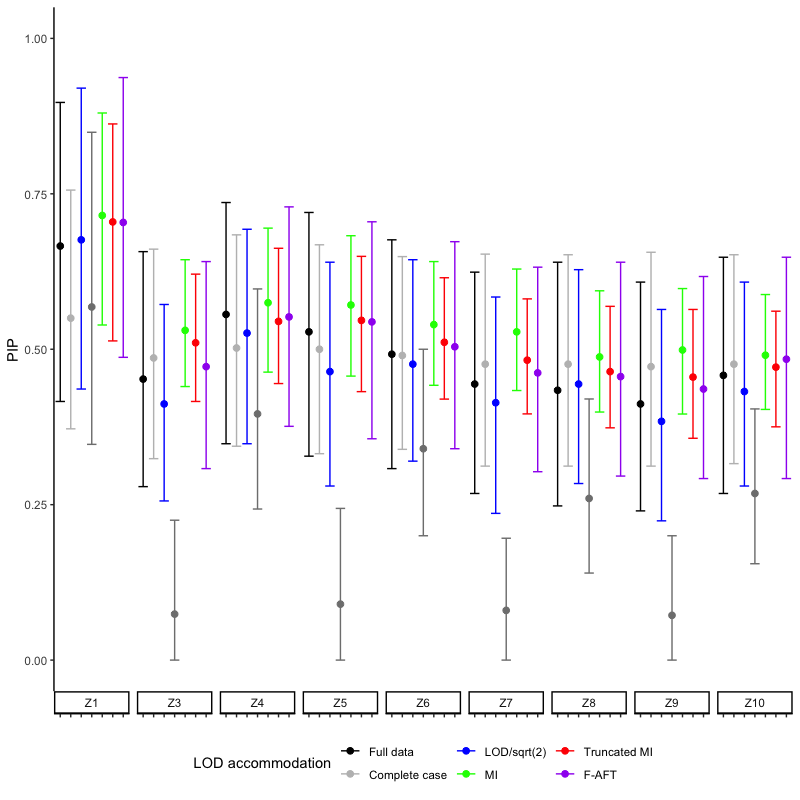


1. **Scenario 2B with moderate correlation setting**


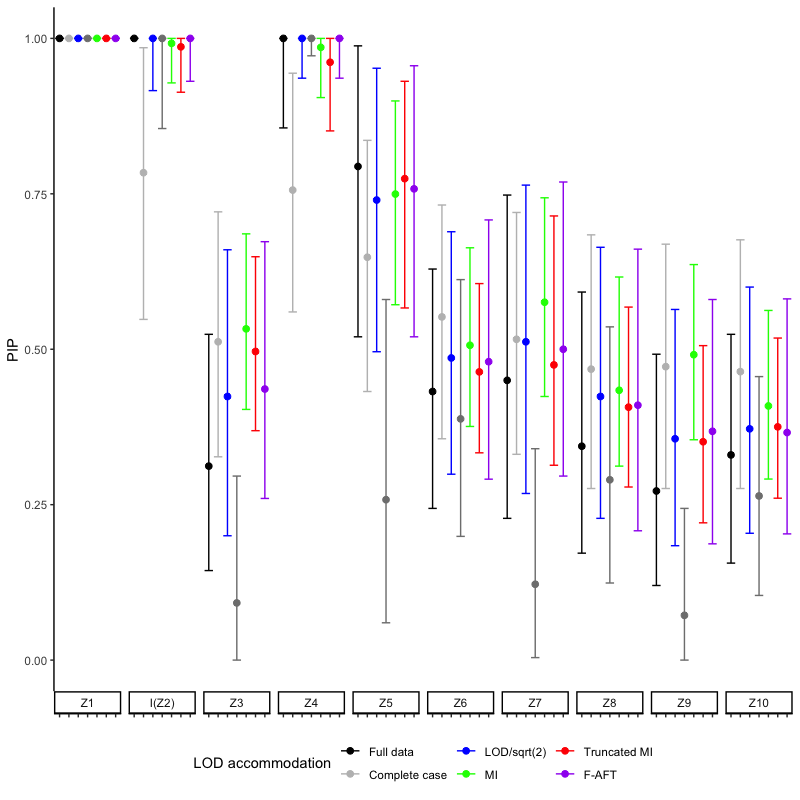


1. **Scenario 2B with high correlation setting**


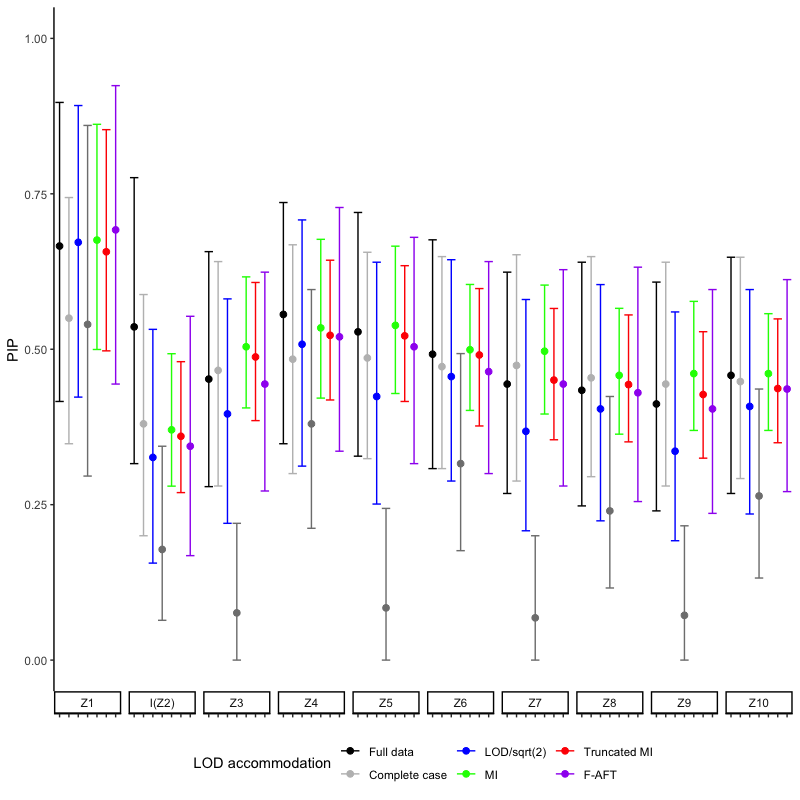


1. **Scenario 3 with moderate correlation setting**


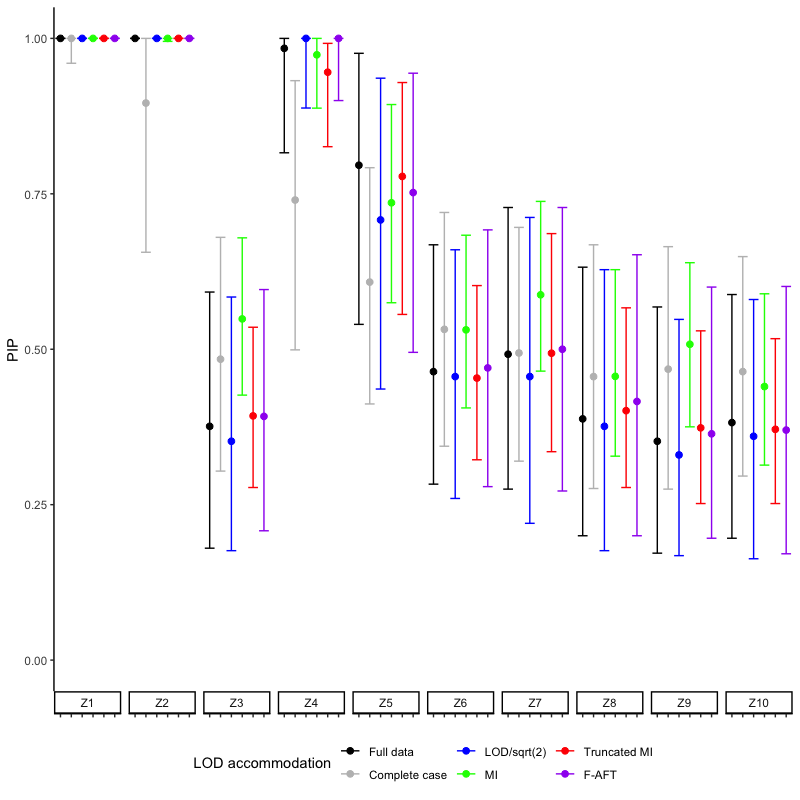


1. **Scenario 3 with high correlation setting**


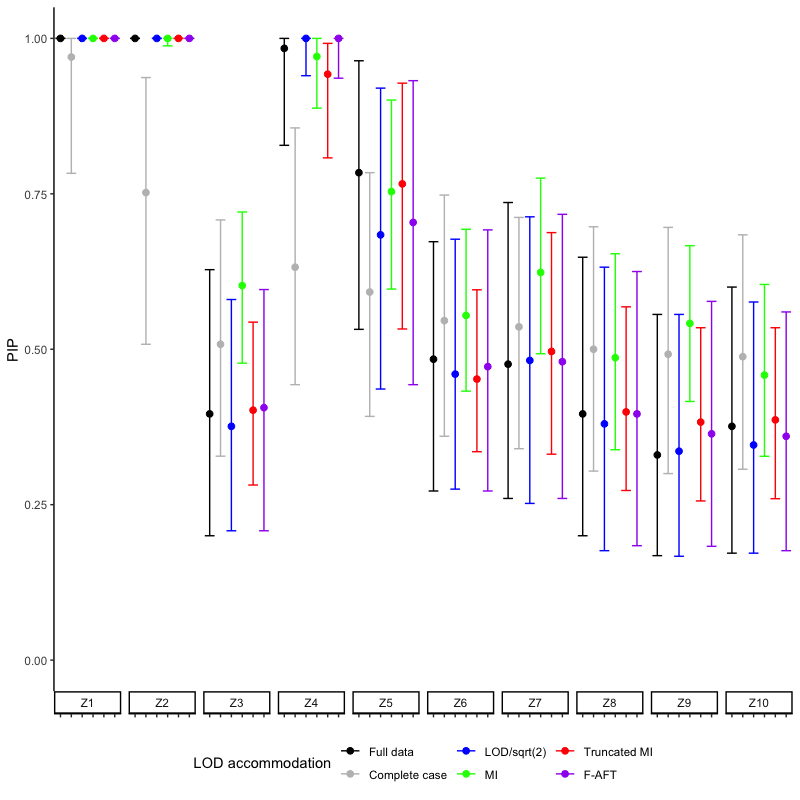


1. **Scenario 4 with moderate correlation setting**


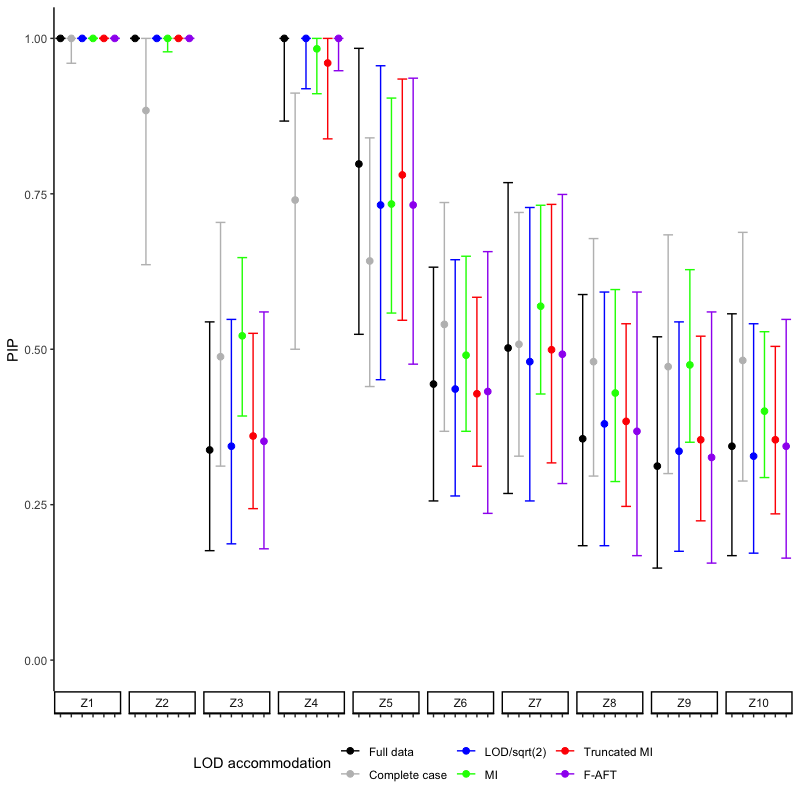


1. **Scenario 4 with high correlation setting**


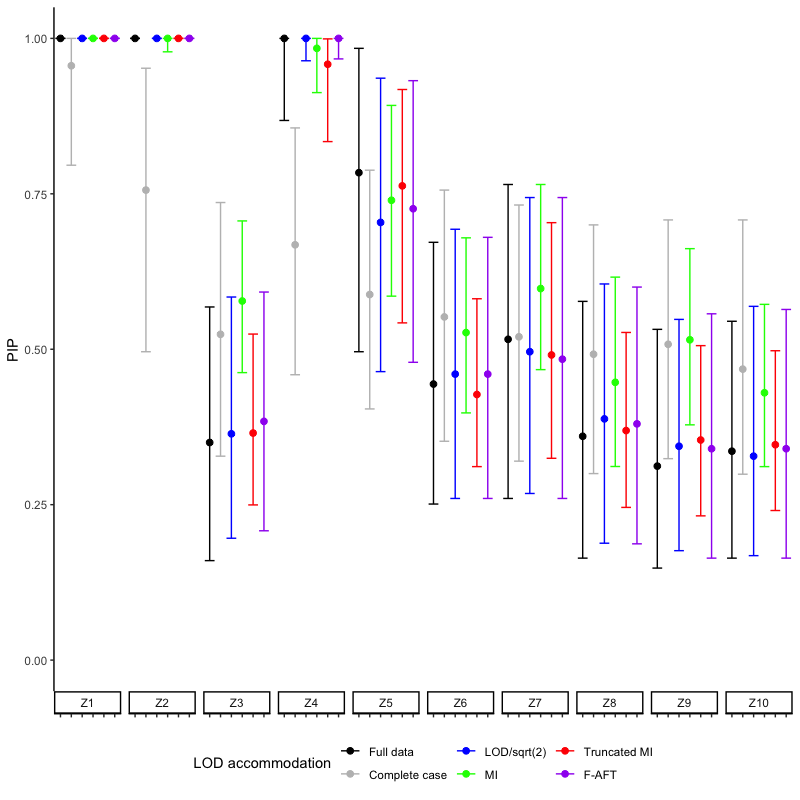


**Abbreviations**: Imputation by LOD/$\sqrt{2}$ (LOD/$\sqrt{2}$); conventional multiple imputation (MI); truncated multiple imputation (Truncated MI); imputation by estimates using the AFT model (F-AFT).

**Figure S3. Coefficients for 18 POPs from group Lasso using NHANES 2001-2002 data.**


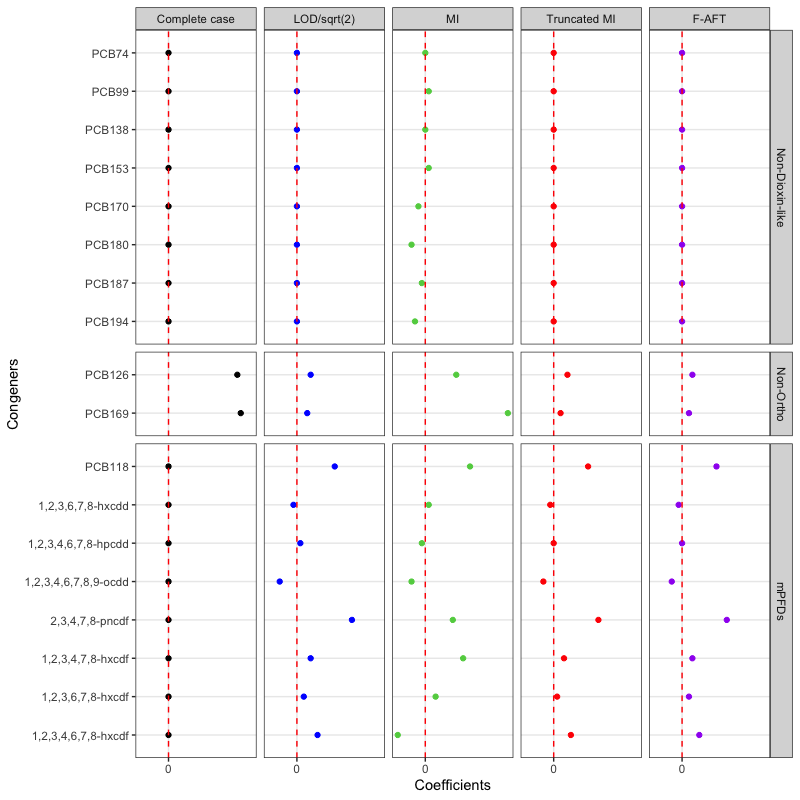


**Abbreviations**: Imputation by LOD/$\sqrt{2}$ (LOD/$\sqrt{2}$); conventional multiple imputation (MI); truncated multiple imputation (Truncated MI); imputation by estimates using the AFT model (F-AFT).
